# Dock2 generates characteristic spatiotemporal patterns of Rac activity to regulate neutrophil polarisation, migration and phagocytosis

**DOI:** 10.3389/fimmu.2023.1180886

**Published:** 2023-06-13

**Authors:** Polly A. Machin, Anna-Karin E. Johnsson, Ellie J. Massey, Chiara Pantarelli, Stephen A. Chetwynd, Julia Y. Chu, Hanneke Okkenhaug, Anne Segonds-Pichon, Simon Walker, Angeliki Malliri, Yoshinori Fukui, Heidi C. E. Welch

**Affiliations:** ^1^ Signalling Programme, The Babraham Institute, Babraham Research Campus, Cambridge, United Kingdom; ^2^ Imaging Facility, The Babraham Institute, Babraham Research Campus, Cambridge, United Kingdom; ^3^ Bioinformatics Facility, The Babraham Institute, Babraham Research Campus, Cambridge, United Kingdom; ^4^ Cell Signalling, Cancer Research UK Manchester Institute, Manchester, United Kingdom; ^5^ Division of Immunogenetics, Department of Immunobiology and Neuroscience, Medical Institute of Bioregulation, Kyushu University, Fukuoka, Japan

**Keywords:** Dock2, guanine-nucleotide exchange factors (GEFs), neutrophils, Prex, Rac, Rho GTPases, Tiam, Vav

## Abstract

**Introduction:**

Rac-GTPases and their Rac-GEF activators play important roles in neutrophil-mediated host defence. These proteins control the adhesion molecules and cytoskeletal dynamics required for neutrophil recruitment to inflamed and infected organs, and the neutrophil effector responses that kill pathogens.

**Methods:**

Here, we used live cell TIRF-FRET imaging in neutrophils from Rac-FRET reporter mice with deficiencies in the Rac-GEFs Dock2, Tiam1 or Prex1/Vav1 to evaluate if these proteins activate spatiotemporally distinct pools of Rac, and to correlate patterns of Rac activity with the neutrophil responses they control.

**Results:**

All the GEFs were required for neutrophil adhesion, and Prex1/Vav1 were important during spreading and for the velocity of migration during chemotaxis. However, Dock2 emerged as the prominent regulator of neutrophil responses, as this GEF was required for neutrophil polarisation and random migration, for migration velocity during chemokinesis, for the likelihood to migrate and for the speed of migration and of turning during chemotaxis, as well as for rapid particle engulfment during phagocytosis. We identified characteristic spatiotemporal patterns of Rac activity generated by Dock2 which correlate with the importance of the Rac-GEF in these neutrophil responses. We also demonstrate a requirement for Dock2 in neutrophil recruitment during aseptic peritonitis.

**Discussion:**

Collectively, our data provide a first direct comparison of the pools of Rac activity generated by different types of Rac-GEFs, and identify Dock2 as a key regulator of polarisation, migration and phagocytosis in primary neutrophils.

## Introduction

Neutrophils are leukocytes important in host defence against bacterial and fungal infections, as well as a host of other functions. They are rapidly recruited from the blood stream into inflamed and infected tissues, through a well-defined cascade of steps, including selectin-dependent tethering and rolling on the vascular endothelium, integrin-dependent arrest, firm adhesion and intravascular crawling, transmigration through the endothelium, and chemotaxis through the interstitium towards the source of inflammation or infection (1, 2). There, the neutrophils release proinflammatory mediators to attract other inflammatory cells and mount a battery of effector functions, including phagocytosis, degranulation, and the production of reactive oxygen species (ROS) and neutrophil extracellular traps (NETs) to kill pathogens (2, 3). The requirement for neutrophils in host defence is evident in neutropenic individuals such as cancer patients undergoing chemotherapy, as well as in conditions like Human Leukocyte Adhesion Deficiency and Neutrophil Immunodeficiency Syndrome (4–7). Yet, excessive recruitment and neutrophil effector responses exacerbate inflammation and cause tissue injury (1, 3, 8).

Rac proteins are small guanine-nucleotide binding proteins (G proteins, GTPases) of the Rho family. Neutrophils express the ubiquitous isoform Rac1, the hematopoietic Rac2 and the more distantly related RhoG (9). These GTPases control the structure of the actomyosin cytoskeleton, enabling the polymerisation of branched actin filaments at the neutrophil’s periphery which is required for adhesion, spreading, and the formation of a leading edge that confers polarisation and migration. Indeed, localised activation of Rac at the leading edge is sufficient to drive directional neutrophil migration (10). In addition to adhesion and migration, Rac-GTPases control essentially all neutrophil effector responses that require cytoskeletal dynamics, including phagocytosis, degranulation, and the production of ROS and NETs (9).

Like most small GTPases, Rac proteins are molecular switches, active when GTP-bound and inactive when GDP-bound (11). They are activated when guanine-nucleotide exchange factors (GEFs) remove GDP, thus allowing excess free cellular GTP to bind to Rac. In this GTP-bound, active conformation, Rac-GTPases engage target proteins that transmit signals downstream, (12, 13), including effectors such as Wave which signals to Arp2/3 to stimulate actin polymerisation (14). Neutrophil Rac-GEFs include proteins from the Prex, Vav, Tiam and Dock families, which each have been assigned roles in neutrophil adhesion or migration (9, 15–18). Prex1^–/–^ neutrophils show reduced actin polymerisation, adhesion and speed of migration upon stimulation with chemoattractants such as formyl-methionyl-leucyl-phenylalanine (fMLP), although their directional sensing is intact (19–21), as well as showing reduced ROS production and killing of *S. pneumoniae* (21–23). Under shear flow conditions, Prex1^–/–^ neutrophils show impaired rolling and intravascular crawling, through reduced functioning of the β2-integrins LFA-1 and Mac-1, respectively (24). *In vivo*, Prex1^–/–^ mice show impaired neutrophil recruitment during sterile peritonitis, ischemia reperfusion injury of the kidney and *S. pneumoniae*-infection of the lung, and their immunity against *S. pneumoniae* is weakened (20, 23–25). Vav1^–/–^ neutrophils show reduced fMLP-stimulated actin polymerisation and chemotaxis, as well as impaired Mac-1 dependent crawling under shear flow conditions (26, 27). Yet surprisingly, neutrophil recruitment is largely normal in Vav1^–/–^ mice in various inflammation and infection models, even when Vav1-deficiency is combined with deficiencies in other Vav GEFs (26–30). Knockdown of Tiam2 in neutrophil-like cells derived from mouse haematopoietic stem cells was shown to inhibit chemotaxis, but curiously increased fMLP-stimulated actin polymerisation and integrin clustering, suggesting that this GEF may limit adhesion rather than promoting it as other Rac-GEFs do (31). We recently used Tiam1^–/–^ neutrophils to show that Tiam1 plays similarly complex roles in β1- and β2-integrin dependent neutrophil responses (manuscript submitted). Dock2^–/–^ neutrophils show impaired chemoattractant-stimulated actin polarisation, leading edge formation and migration speed, whereas β2-integrin mediated adhesion and directional sensing were normal (32, 33). *In vivo*, Dock2 is required for neutrophil recruitment to the colonic mucosa of mice infected with the enteric pathogen *Citrobacter rodentium* (34). A human loss-of-function mutation in DOCK2 results in severe immunodeficiency with impaired neutrophil actin polymerisation, spreading and ROS production (35). We recently conducted a genome-wide association study which identified a risk allele associated with decreased DOCK2 expression in patients with severe COVID-19, and we showed that blockade of DOCK2 activity with the small molecule inhibitor CPYPP increased the severity of pneumonia in a Syrian hamster model of SARS-CoV-2 infection (36).

Altogether, the different neutrophil Rac-GEFs all regulate neutrophil adhesion and migration, with more or less subtle differences between them. This raises the question why so many different Rac-GEFs are required within the same cell type. One reason is that the GEFs show substrate preferences for different Rac isoforms. For example, Prex1 prefers to activate Rac2 over Rac1 in response to fMLP stimulation, whereas Vav GEFs do the inverse (20, 21). Some Rac-GEFs can also activate other Rho-GTPases, for example Vav1 can activate RhoA as well as Rac (16). Another reason is that the Rac-GEFs couple to different upstream regulators. Rac-GEF activity is tightly regulated, and the mechanisms of regulation have often been studied in minute detail. In addition to their catalytic domains, Rac-GEFs comprise multiple and varied domains that couple each GEF to specific regulators. It is assumed, and in some instances proven, that integration of these regulatory mechanisms determines precisely which Rac-dependent cell response ensues. Yet, within the neutrophil, there is considerable overlap between the surface receptors and signalling pathways that the different types of Rac-GEFs couple to (9, 37, 38).

In contrast to GEF regulation, we understand much less whether the different GEFs generate distinct pools of Rac activity within the neutrophil, in space and time. This gap in knowledge is in part due to the nature of the neutrophil, a short-lived cell (hours) in which protein expression is difficult to manipulate. Neutrophil-like cell lines are generally poor substitutes, so alteration of gene expression in primary neutrophils is mostly done using mouse genetics. The complexity of mouse genetics means direct comparisons between Rac-GEFs have rarely been undertaken, so it is unclear how large their functional differences are. Another factor that has hampered the identification of specific functions for each GEF is redundancy. The few studies which conducted direct comparisons between neutrophil Rac-GEFs revealed several examples of redundancies, within the same GEF family or between families. For example, Vav1^–/–^ Vav3^–/–^ neutrophils show defects in Fc receptor-dependent and integrin-dependent adhesion and spreading, which are not seen in cells lacking either Vav isoform alone (28, 39). Similarly, deficiency in Dock5 has little effect on its own, but combined deficiency with Dock2 exacerbates the migration defect caused by the absence of Dock2 (40). We previously showed that Prex1 and Vav1 cooperate in a range of neutrophil responses, including integrin-dependent adhesion and spreading, and in neutrophil recruitment to the inflamed peritoneum or lung (21, 25).

To address this gap in knowledge, we established a system that enables us to compare different Rac-GEFs directly in primary neutrophils. We previously generated a reporter mouse strain for Rac activity, Rac-FRET (41), which expresses a Raichu-Rac FRET reporter originally created by the Matsuda lab (42). Here, we crossed the Rac-FRET reporter mouse with strains deficient in Dock2, Tiam1 or Prex1/Vav1 to evaluate if these Rac-GEFs activate spatiotemporally distinct pools of Rac, and to correlate these patterns of Rac activity with the neutrophil responses they control. Our study identifies Dock2, above the other Rac-GEFs, as a key regulator of neutrophil polarisation, random migration, chemokinesis and phagocytosis, and reveals the specific underlying spatiotemporal patterns of Rac activity generated by this GEF.

## Materials and methods

### Mice

The Rac-FRET reporter mouse strain for Rac activity, which ubiquitously expresses an intramolecular Raichu-Rac FRET probe for Rac activity, was previously described (41). This reporter strain was crossed to various Rac-GEF deficient strains, namely Dock2^–/–^ (43), P-Rex1^–/–^ Vav1^–/–^ (21) and Tiam1^–/–^ (44). All strains were on C57BL/6 genetic background. Mice were bred and group-housed (up to 5) in specific opportunistic pathogen-free (SOPF) isolators in a shower-in SOPF barrier facility of the Babraham Institute Small Animal Facility that uses 12 h light/dark cycles with dusk and dawn settings, 52% room humidity (55 ± 10% range) and 20°C room temperature (19-21°C range), and were fed chow diet and water *ad libitum*. The SOPF status of the unit is monitored by quarterly testing of sentinels for 62 pathogens, exceeding current FELASA guidelines (45). Staff work in designated units, with showering-in, wearing of fresh autoclaved uniforms, gloves, hairnets and masks, and with a 48 h exclusion from other units. All materials are autoclaved or treated with vaporised hydrogen peroxide on import. The animal diet is sterilised to ≥25 Gy. Cages are opened in laminar flow cabinets. Genotyping was carried out by Transnetyx (Memphis, USA). Male and female mice were used between 8‐14 weeks of age, and were sex- and age‐matched in experiments as far as possible. Animal breeding and experiments were carried out with approval from the local Animal Welfare Ethical Review Body under the British Home Office Animal Scientific Procedures Act 1986.

### Neutrophil purification

Mature primary neutrophils were freshly isolated each day from mouse bone marrow by Percoll^PLUS^ gradient at 4°C using endotoxin-free reagents throughout, essentially as previously described (23, 46). Briefly, mouse bone-marrow was flushed from femurs, tibias and pelvic bones with ice-cold Hank’s Balanced Salt Solution (HBSS) without Ca^2+^ or Mg^2+^ (HBSS^–^, Sigma H6648) supplemented with 15 mM HEPES, pH 7.4 (RT) (Sigma, H3784) and 0.25% fatty acid-free (FAF) BSA (Sigma, A8806) (HBSS^–++^), triturated and filtered through a 40 μm cell strainer. 58% isotonic Percoll^PLUS^ (GE Healthcare, 17544501) in HBSS^–++^ was underlayed and samples were spun at 1620 × g without brake for 30 min at 4°C. The lower 5 ml of the gradient were resuspended in 40 ml HBSS^–++^ and centrifuged at 326 × g for 10 min at 4°C. Erythrocytes were lysed in Geye’s solution (130 mM NH_4_Cl, 5 mM KCl, 780 µM Na_2_HPO_4_, 176 µM KH_2_PO_4_, 5.5 mM glucose, 1 mM MgCl_2_, 280 µM MgSO_4_, 1.54 mM CaCl_2_, 13.4 mM NaHCO_3_) for 3 min at RT. 10 volumes of ice-cold HBSS^–++^ were added and cells sedimented again. Neutrophils were resuspended in ice-cold Dulbecco’s Phosphate Buffered Saline (DPBS) with Ca^2+^ and Mg^2+^ (DPBS^++^, Thermo Fisher Scientific, 14040117) supplemented with 0.1% glucose and 4 mM NaHCO_3_ (DPBS^++++^) and were kept on ice while aliquots were counted by haemocytometer and purity assessed by Kwik-Diff stain (Thermo Scientific Shandon, 9990700) of cytospins. Preparations were >90% pure. Neutrophils were sedimented again and resuspended in the buffer appropriate for the subsequent assay.

### Adhesion, spreading and polarisation (fixed cells)

To measure neutrophil adhesion, spreading and polarisation on ICAM1, Ibidi 8-well glass-bottom μ-slides (Ibidi, 80827) were coated overnight at RT with 3 µg/ml ICAM1 (Fc-chimera, R&D Systems, 796-1C), then blocked in 2% (w/v) BSA (Sigma, A7906) in DPBS^++^ for 1 h, and washed 3 times in DPBS^++^ before use. Isolated neutrophils were resuspended at 4.5 x 10^6^/ml in DPBS^++++^ and primed with 50 ng/ml GM-CSF (Peprotech, 315-03) and 20 ng/ml TNFα (R&D Systems, 410-MT-010) for 45 min at 37°C. The coated Ibidi slides were pre-warmed to 37°C, and 150 µl of primed neutrophils were added to 150 µl of pre-warmed 1.5 µM (2x) fMLP (Sigma, F3506) in DPBS^++++^ and were allowed to adhere for 1 h, before being fixed in 4% paraformaldehyde (PFA, Sigma, P6148, in DPBS) for 10 min on ice and stained with FTIC-Gr1 antibody (BD Biosciences, 553126, clone RB6-8C5, 1:800), phalloidin-Atto 655 (Sigma, 18846), and Hoechst 33342 DNA dye (Thermo Fisher, 62249, 1:400). Samples were imaged by wide-field fluorescence microscopy on a Nikon Eclipse Ti-E widefield microscope. Images were analysed using Fiji (ImageJ) software (47), by drawing a mask for each neutrophil. To quantify neutrophil adhesion, the number of neutrophils/FOV was determined, with particles smaller than 40 μm^2^ being excluded from the analysis. Spreading and polarity were quantified as the surface area and circularity of the mask, respectively using the ‘Set Measurements’ analysis tools of Fiji.

To measure neutrophil adhesion and spreading on glass, assays were performed essentially as described (21, 23). Purified neutrophils were resuspended at 2 × 10^6^/ml in DPBS^++++^ and primed with 20 ng/ml TNFα and 50 ng/ml GM-CSF for 45 min at 37°C. 250 μl cells were added onto sterile 13 mm glass coverslips (VWR, 631) in a 24-well plate (Thermo Fisher, Nunc 142475) containing 250 μl of 1.5 μM (2x) fMLP in DPBS^++++^, or DPBS^++++^ alone, and were incubated for 10 or 25 min at 37°C in a humidified, 5% CO_2_ incubator. Non-adherent cells were aspirated and adherent cells fixed in 4% PFA for 15 min at RT. Cells were washed twice in PBS and stained with FITC-Gr1 antibody and Hoechst 33342 DNA dye in PBS, 1% Fc block (BD Biosciences, 553141), for 30 min at RT. Coverslips were washed three times in PBS, rinsed in H_2_O and mounted onto slides with ProLong Gold Antifade (Life Technologies, P36934). Imaging was done using the ‘large image capture’ function of a Nikon Eclipse Ti-E widefield system, taking 27 (3 x 9) fields-of-view (FOV) per coverslip at 100x magnification, and duplicate coverslips per condition. Image analysis was done using Fiji as described here-above.

### TIRF-FRET imaging of Rac activity

Ratiometric TIRF-FRET microscopy was used in live neutrophils to visualise and quantify the spatiotemporal distribution of Rac activity at the basal cell surface during adhesion, spreading, polarisation, migration and phagocytosis. Isolated neutrophils were plated on various surfaces at 37°C, as detailed for each cell response, inside a temperature-controlled microscope environment chamber. Neutrophils of the various genotypes were directly compared within each experiment, and were imaged each time in alternating order. To determine Rac activity, ratiometric TIRF-FRET live imaging was performed by taking frames either every 1 or 5 s, as specified. A Nikon Ti2 TIRF microscope with 60x 1.49 NA oil TIRF objective was used with laser excitation at 440 nm. An Andor TuCam emission beam splitter comprising 509 nm dichroic mirror, 480/40 nm (CFP) and 542/27 nm (YFP) band-pass filters (Semrock) was used to direct the fluorescence from the donor channel (CFP) and FRET channel (YFP) to Andor iXon 897 EM-CCD cameras. Channel alignment was performed at the start of each experiment using the Fiji plugin bUnwarpJ (48), by creating a transformation protocol for images taken of CPN™ 530 Green standard beads (Stream Bio), which fluoresce in both the CFP and YFP channels. This transformation protocol was then applied to the whole series of CFP and YFP image pairs. To ensure that CFP and YFP channel images were taken simultaneously, an automated external trigger for the ‘slave’ camera was connected to the ‘master’ camera. Once CFP and YFP image pairs were aligned, a mean filter of two pixels was applied and the YFP/CFP ratio image generated as described (41). Ratiometric TIRF-FRET images were depicted using the Fiji 16-colour table, pseudo-colouring high Rac activity (high FRET ratio) in red and low Rac activity (low FRET ratio) in blue.

### Adhesion and spreading (live cells)

To prepare the surfaces for adhesion and spreading assays, Ibidi 8-well glass-bottom μ-slides (Ibidi, 80827) were coated overnight at RT with 20 µg/ml poly-Arg-Gly-Asp (pRGD, Sigma, F5022) or with 3 µg/ml ICAM1 (Fc-chimera, R&D Systems, 796-1C). ICAM1-coated slides were additionally blocked in 2% (w/v) BSA (Sigma, A7906) in DPBS^++^ for 1 h. Alternatively, where CD18 antibody and ICAM1 coating were compared, slides were incubated with 100 µg/ml poly-D-lysine (Sigma, P6407) and then with 25 μg/ml protein A (Sigma, P6031) for 1 h at RT, washed, and 10 μg/ml activating mouse CD18 antibody (eBioscience, 14-0181-81, clone M18/2) or 3 µg/ml ICAM1 were added for 1 h at 37˚C, before slides were washed again and blocked in BSA. All slides were washed 3 times in DPBS^++++^ before use. Isolated neutrophils were resuspended at 4.5 x 10^6^/ml in DPBS^++++^ and primed with 50 ng/ml GM-CSF and 20 ng/ml TNFα for 45 min at 37°C. To measure Rac activity during adhesion and spreading, the coated Ibidi slides were pre-warmed to 37°C in the imaging chamber. 150 µl of primed neutrophils were added to 150 µl of pre-warmed 2x fMLP (1.5 µM) or KC (100 ng/ml) in DPBS^++++^, or DPBS^++++^ alone, in the imaging well, and live-cell ratiometric TIRF-FRET imaging was performed as described here-above. To measure the initial spreading, neutrophils were imaged for 200 s, with frames taken every 1 s, to capture Rac activity from the moment the cell made first contact with the surface. To measure Rac activity during firm adhesion, the cells were allowed to adhere for 1 h, in the presence or absence of fMLP, before being imaged by live-cell ratiometric TIRF-FRET microscopy for 130 s, with frames taken every 5 s. To quantify changes in overall Rac activity at the basal cell surface that touches the substrate, the mean FRET ratio (pixel intensity value) for the whole cell was plotted against time.

### Polarisation and random migration (live cells)

Neutrophil polarisation and random migration were tested in live cells essentially as previously described (41), combined with TIRF-FRET imaging of Rac actvity. Briefly, isolated neutrophils were resuspended in HBSS^++^ at 37˚C, plated onto sterile glass coverslips in the environment chamber and imaged by live-cell ratiometric TIRF-FRET imaging as described above, in order to quantify Rac activity at the basal cell surface during neutrophil polarisation. Imaging commenced as soon as the cells settled onto the coverslip, from the first point of contact, and was done over 10 min, with frames taken every 5 s. The mean Rac activity over the entire basal cell surface was calculated for each time point as described above. In addition, line scans were performed along the central longitudinal axis of the neutrophils for each frame to examine the spatial distribution of Rac activity between leading edge and uropod over time, and this Rac activity was grouped into 20 bins along the length of the cell, as described (41). The basal cell surface area was determined using a mask in Fiji, as described for the spreading assay. In addition, the time until cells started forming a first leading edge protrusion, the number of protrusions per cell, and the proportion of cells undergoing random migration, with displacement of at least one cell diameter over the 10 min observation period, were assessed manually, as previously described (41).

### Chemokinesis (ibidi)

Chemokinesis assays were performed essentially as described (46), combined with TIRF-FRET imaging of Rac actvity. Isolated neutrophils were resuspended at 4.5 x 10^6^/ml in DPBS^++++^ and primed with 50 ng/ml GM-CSF and 20 ng/ml TNFα for 45 min at 37°C. The primed cells were allowed to adhere to an ICAM1-coated ibidi μ-slide (as described above) for 5 min at 37˚C in the environment chamber, in a bath application of 0.75 µM fMLP, prior to the start of imaging. Migrating cells were imaged by live-cell ratiometric TIRF-FRET microscopy for 2 min, with a frame interval of 1 s, to enable analysis of Rac activity during chemokinesis. The cells were analysed for migration speed by measuring the position of the front pixel of the cell over 20 s, for cell area using a mask as described above, and for mean Rac activity over the basal cell surface of the whole cell. The latter was determined as described above for the live-cell adhesion assay. Cell area and Rac activity were determined for actively migrating phases and stalling phases of migration separately, which we defined as periods of at least 20 s duration where the cell either did or did not actively protrude a leading edge lamella.

### Chemotaxis (transwell)

Transwell chemotaxis assays were done essentially as described (46) using 3 μm-pore polycarbonate filters (Millipore, Millicell-PC, PITP01250) in ultra-low cluster 24-well tissue culture plates (Costar, 3473). Bone marrow was flushed into HBSS with Ca^2+^ and Mg^2+^ (Sigma, H8264), supplemented with 0.25% fatty acid-free BSA, and 15 mM Hepes, pH 7.5 at 37°C, all endotoxin-free (HBSS^++++^), triturated, filtered through a 40 μm nylon cell strainer, counted by haemocytometer and adjusted to 5 x 10^6^/ml. 400 μl cells were pipetted into each transwell filter in a 24-well plate containing 600 μl HBSS^++++^ per well in the presence or absence of 3 μM fMLP, and were incubated for 40 or 90 min at 37°C. Cells remaining in the transwell were removed and replaced with 400 μl ice-cold HBSS^–++^ containing 3 mM EDTA. 60 µl HBSS^–++^ containing 30 mM EDTA was added to the bottom well, and plates were incubated on iced metal trays for 15 min to detach cells. Transmigrated cells were collected and, in parallel to control cells, were centrifuged at 10,000 x g for 30 s and resuspended in ice-cold HBSS^–++^. Cells were stained with FITC-Gr1 (clone RB6-8C5, BD Pharmingen, 553126, 1:800) and AF647-CD11b (clone M1/70, BD Pharmingen, 557686, 1:800) antibodies in HBSS^–++^ containing 1% Fc block and were analysed using an LSR2 flow cytometer alongside Spherotech ACBP-50-10 standard beads. Neutrophils were identified by their Gr1^hi^/CD11b^hi^ staining using FlowJo software. Transmigrated neutrophils were compared to total neutrophils in control samples.

### Chemotaxis (micropipette)

Live-cell micropipette chemotaxis assays were performed essentially as previously described (49, 50), combined with TIRF-FRET imaging of Rac actvity. Briefly, isolated neutrophils were resuspended in HBSS^++++^ at 1.25 × 10^5^/ml and allowed to attach to a glass coverslip at 37°C in a temperature-controlled microscope environment chamber (Solent Scientific). Cells were stimulated with a point source of 0.5 μM fMLP delivered from a femtotip microinjection needle (Eppendorf, 5242957008) originally placed ~20 μm from the cell, using 50 mbar back pressure. In some experiments, the needle was moved to a position 90˚ anti-clockwise half-way through the experiment, to measure the ability of the cell to turn with the chemoattractant gradient. To quantify Rac activity during neutrophil chemotaxis towards the micropipette, cells were imaged for 2 min by live-cell ratiometric TIRF-FRET imaging, as described above, with a frame interval of 1 s. To plot the intensity and distribution of Rac activity around the periphery of the migrating cell over time, cells were tracked using Fiji plugin QuimP11 (www.warwick.ac.uk), to determine FRET ratios within 0.4 μm of the outer edge for each frame (41) over time. Polar plots were derived by mapping the Rac-FRET signal along the cell circumference for each frame onto a circle for individual cells, and combining sequential frames as eccentric circles, with the first frame in the centre and the last at the plot edge (41, 49). Plots were aligned so that the fMLP-containing micropipette was positioned due west first, and then south where the needle was moved half-way through the experiment to investigate turning. Plots from all cells of the same genotype were combined to depict mean Rac activity around the cell periphery. The movement plot function of Fiji plugin QuimP11 was used to plot the path migrated. Additionally, the time it took for neutrophils to form the first leading edge protrusions after the micropipette was moved to a new location was measured. To determine the front/back polarity of Rac activity in the chemotaxing neutrophils, line scans were performed through the central longitudinal axis of the cell for each frame. To compare the height and localisation of Rac activity, migrating and stationary cells were analysed separately, with migrating cells defined as those that translocated at least 7.8 μm (half their mean body length), during the 2 min observation period. Mean Rac-FRET ratios of 5% bins over the cell length were plotted to give 20 data points along the longitudinal axis of each cell, from the leading edge to the uropod, as described above for the polarisation assay. Kymographs plotting these longitudinal axes of Rac activity as a function of time were generated as described (41). In addition, the pixel with the highest Rac-FRET ratio along the longitudinal axis was determined for each frame. The time that peak Rac activity was localised within 0.8 μm of the leading edge or uropod throughout the 2 min observation period was plotted, and the number of times that peak Rac activity oscillated between leading edge and uropod was determined as previously described (41).

### Phagocytosis (fixed cells)

2 µm Fluoresbrite^®^ yellow/green carboxylate microspheres (Polysciences, 098475) were coated with BSA by incubation with 1 mg/ml BSA in 50 mM MES buffer (pH 6.7) containing 20 mg/ml EDAC (Sigma, E7750) for 1 h at RT, washed, and stored in 10 mM Tris pH 8, 0.05% BSA. To opsonise the beads with IgG, they were washed in PBS, incubated with anti-BSA IgG (1:250 in PBS) for 1 h at RT, washed, and resuspended in HBSS^++++^. Alternatively, beads remained unopsonised. Purified neutrophils were resuspended at 1x10^7^/ml in HBSS^++++^ and primed with 20 ng/ml TNFα and 50 ng/ml GM-CSF for 45 min at 37°C as described above. 100 μl of cells were combined with 100 μl BSA-coated or IgG-opsonised beads at 1 x 10^8^/ml (10 particles/neutrophil) in a 24-well plate containing sterile glass coverslips, and were incubated for 15 min at 37˚C. Cells were fixed with 4% PFA in for 15 min and stained with FITC-Gr1 (BD Pharmingen, 553126, 1:800) containing 1% Fc block and mounted in ProLong Gold Antifade mountant (Life Technologies). Image analysis was done with Fiji, using the FITC-Gr1 signal at the phagosomal membrane to distinguish phagosomes from particles attached to the outside of the cell. The number of particles taken up per cell, and the percentage of neutrophils containing at least one particle were enumerated manually.

### Phagocytosis (live cells)

To measure phagocytosis of IgG-opsonised beads in live neutrophils, particles were prepared and cells treated as described here above, except that cells were plated on glass coverslips in the 37°C environment chamber and live-imaged by TIRF-FRET microscopy as described above, to allow imaging of Rac activity during phagocytosis. Imaging was done for 15 min, with frames taken every 5 s, and using brightfield imaging in parallel to visualise cells and particles at the same time. To quantify Rac activity around the phagosomal membrane, mean FRET ratio in the area of the forming phagosome was evaluated from the moment the cell made contact with a particle until the particle was fully engulfed. In addition, the time taken for full engulfment was plotted.

### Peritonitis

Aseptic peritonitis was induced as previously described (46). 9-10 week-old male mice were injected *i.p.* with 0.25 ml sterile 3% thioglycollate (TGC, Sigma, T9032) in H_2_O, or were mock-treated with H_2_O, before being returned to their home cages with food and water *ad libitum*. 3 h later, mice were euthanized by CO_2_ asphyxiation, death confirmed by pithing, and peritoneal lavages performed by *i.p.* injection and aspiration of 8 ml DPBS^–^, 5 mM EDTA. A second lavage was performed, pooled with the first, and samples stored on ice. The lavage cells were pelleted at 450 x g for 10 min at 4°C, erythrocytes lysed by resuspending cells in 1 ml Geye’s solution and incubating at RT for 150 s, prior to the addition of 10 ml DPBS^++++^. Leukocytes were resuspended in 1.25 ml DPBS^++++^. 1 ml of the sample was processed for flow cytometry by staining leukocytes with AF647-Cd11b (clone M1/70, BD Pharmingen, 557686, 1:800) and FITC-Gr1 (clone RB6-8C5, BD Pharmingen, 553126, 1:800) antibodies in DPBS^++++^ with 1% Fc block for 20 min on ice in the dark, washing in DPBS^++++^, 5 mM EDTA and resuspension in 500 μl DPBS^++++^ containing 1 μg/ml DAPI and 1.25 × 10^5^ Spherotech ACBP-50-10 standard beads/ml (5.0-5.9 μm). Flow cytometry was carried out in a BD Biosciences LSRII flow cytometer, and FlowJo was used for data analysis. Neutrophils were identified by Cd11b^hi^, Gr1^hi^ staining and enumerated by taking into account the lavage volume recovered. Alternatively, mice were treated with TGC, or mock-treated, as above, culled 3 h later, and peripheral blood was collected by cardiac puncture into EDTA-coated microvettes (Sarstedt, CB 300). One portion was analysed for the number of neutrophils in peripheral blood, by lysing erythrocytes using Geye’s solution as detailed for neutrophil purification, followed by staining and enumeration of neutrophils by flow cytometry as detailed here above. Blood plasma was prepared from the other portion by two centrifugations at 2,000 x g for 5 min at RT, and was analysed for the levels of CXCL1, IL6 and TNFα using DuoSet ELISA kits (R&D Systems DY06, DY453 and DY410, respectively), according to the manufacturer’s instructions.

### Data collection and statistical analysis

Sample size was determined using power calculations to yield 80% power, based on results of pilot experiments and on previously published data as referenced. Within the parameters of age and sex, mice were selected for cohorts at random by the staff of the Small Animal Unit. Image analysis was performed in a blinded manner wherever possible. Excel 2016 and GraphPad Prism 9.0 were used for tabulation, statistical analysis and plotting graphs. Data were tested for normality of distribution using Shapiro-Wilk test to determine if parametric or non-parametric statistical analysis was required. Where warranted by variance between groups, data were log-transformed prior to statistical analysis. Statistical outliers were identified using Tukey’s test and removed from datasets. Other samples were only excluded when there was a known technical problem affecting the analysis. Data were analysed by one-way or two-way ANOVA to test for effects of interventions. Experiments were performed at least three times except where indicated. Sample size and numbers of independent experiments are detailed in figure legends. Effect size and variance are reported as group mean ± standard error. P-values reported are from multiplicity-adjusted Sidak’s or Tukey’s *post-hoc* comparisons, following GraphPad recommendations. The threshold for statistical significance was set at p<0.05. Significant differences are denoted in the figures using black p-values, and in some instances relevant lack of difference is denoted by grey p-values.

## Results

**Figure 1:**
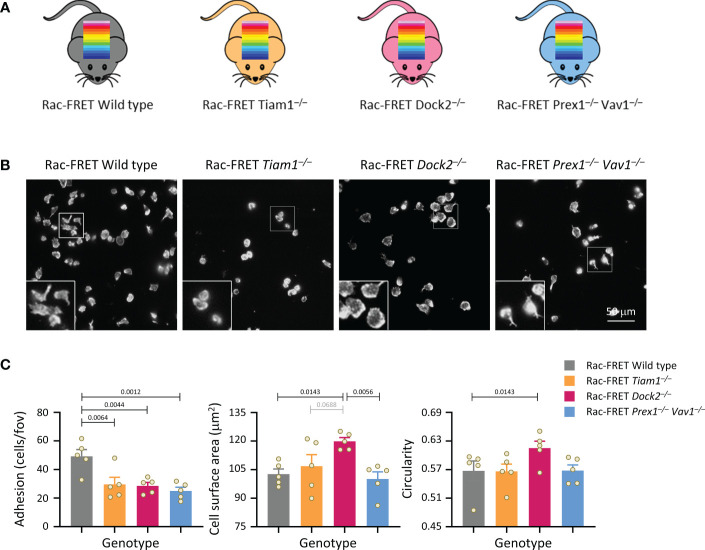
Tiam1, Dock2 and Prex1/Vav1 are required for sustained neutrophil adhesion to ICAM1. **(A)** Schematic of mouse strains used in this study. Rac activity FRET reporter mice (Rac-FRET Wild type, grey), were crossed to mouse strains deficient in the Rac-GEFs Tiam1 (orange), Dock2 (pink) or Prex1/Vav1 (blue). **(B, C)** Requirement for different Rac-GEFs in neutrophil adhesion, spreading and polarisation on ICAM1. Neutrophils from wild type or Rac-GEF deficient Rac-FRET mice as in **(A)** were primed with 50 ng/ml GM-CSF and 20 ng/ml TNFα for 45 min at 37˚C before being plated onto ICAM1-coated slides and incubated for 1 h at 37°C in the presence of 0.75 μM fMLP. Adherent cells were fixed, stained with FTIC-Gr1 antibody, far-red-phalloidin and Hoechst DNA dye, and were imaged by wide-field fluorescence microscopy. **(B)** Representative images from one experiment; the phalloidin staining is shown. Inserts are magnifications of the indicated areas. **(C)** Images were analysed by Fiji for the number of adhering cells (left-hand panel), their surface area (middle) and their circularity (right). Data are mean ± SEM of 5 independent experiments; each dot represents the mean of one experiment. Statistics are one-way ANOVA with Tukey’s multiple comparison corrections.

We sought to dissect the roles played by various Rac-GEFs in neutrophil adhesion and migration, by correlating the spatiotemporal patterns of Rac activity they generate with the cell responses they regulate. To enable this study, we crossed a Rac activity reporter mouse strain (Rac-FRET) which we previously generated (41), to mouse strains with deficiencies in the neutrophil Rac-GEFs Prex1, Vav1, Dock2 or Tiam1 (**Figure 1A**). We previously established that Prex1 and Vav1 cooperate in generating Rac activity and in mediating neutrophil adhesion, spreading, ROS production and *in vivo* recruitment (21, 25), so we combined deficiency in these two GEFs in one Rac-FRET Prex1^–/–^ Vav1^–/–^ mouse strain.

### Rac activity at the basal cell surface does not correlate with the ability of neutrophils to adhere and spread

Initially, we performed simple experiments to analyse neutrophil adhesion, spreading and polarity (without measuring Rac activity), by plating isolated neutrophils from the various Rac-FRET mouse strains onto ICAM1. Neutrophils adhere to ICAM1 through their β2-integrins LFA1 and Mac1, after these integrins are activated by GPCR-dependent inside-out signalling (51). So we plated the cells in the presence of the chemoattractant fMLP to activate the integrins and to stimulate polarisation, and for 1 h to establish steady-state conditions. Adhesion was reduced by half in each GEF-deficient strain, meaning that all GEFs investigated are required for neutrophil adhesion (**Figures 1B, C**). Dock2 deficiency additionally caused neutrophils to spread more and be less polar (more circular), whereas the other GEFs had no effect on spreading and polarity under these conditions (**Figures 1B, C**). Hence, Dock2 suppresses neutrophil spreading and is required for neutrophil adhesion and polarisation on ICAM1. Similar experiments with Rac-FRET Prex1^–/–^ Vav1^–/–^ and Rac-FRET Dock2^–/–^ neutrophils plated onto glass instead of ICAM1 showed that the GEF requirement in neutrophil adhesion and spreading is context-dependent. Dock2 and Prex1/Vav1 were dispensable for adhesion to glass, both constitutively and upon stimulation with fMLP, but Prex1/Vav1 were required for spreading, whereas Dock2 had no effect (**Supplementary Figure 1**). Together, these experiments showed that the Rac-GEFs, Tiam1, Dock2 and Prex1/Vav1 play different, substrate-dependent roles in neutrophil adhesion, spreading and/or polarisation.

**Figure 2:**
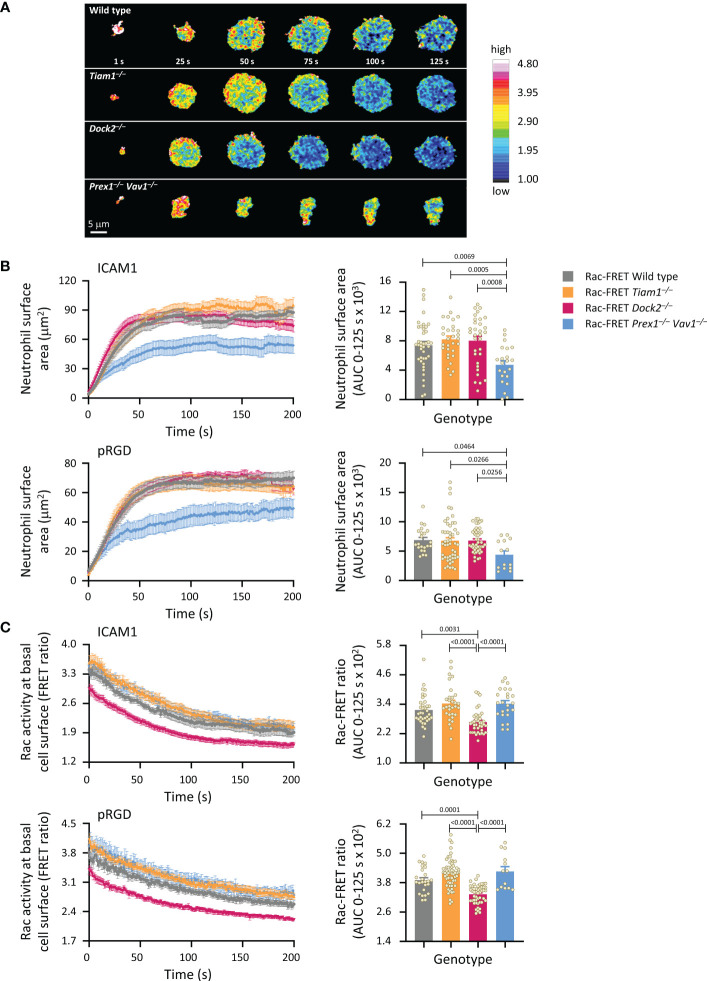
Prex1/Vav1 are required for the initial spreading of neutrophils on integrin ligand surfaces, although Dock2 confers Rac activity during this process. Neutrophils from wild type Rac-FRET mice (grey symbols), or Rac-FRET mice deficient in Tiam1 (yellow), Dock2 (pink), or Prex1/Vav1 (blue) were primed with 50 ng/ml GM-CSF and 20 ng/ml TNFα for 45 min at 37˚C and plated onto ICAM1-coated or pRGD-coated ibidi slides, as indicated, in the presence of 0.75 µM fMLP, and were imaged by live-cell ratiometric TIRF-FRET microscopy from the first point of contact with the slide for 200 s, at a frame interval of 1s. **(A)** Stills of representative movies from one experiment with ICAM1-coated slides. The pseudo-colour scale depicts high Rac activity (high FRET ratio) in red and low Rac activity in blue. **(B)** Cell spreading. Left-hand panels: cell spreading (basal cell surface area) on ICAM1 or pRGD over time. Data are mean ± SEM of neutrophils pooled from 4 independent experiments for ICAM1 and 5 for pRGD, except Prex1^–/–^ Vav1^–/–^ cells which are from 3 experiments. Cells of different genotypes were compared directly within experiments. Right: Quantification of spreading as area under the curve (AUC; mean ± SEM) in the first 125 s of the response. **(C)** Rac activity. Left: Rac activity (FRET ratio, mean ± SEM) at the basal surface of the cells in **(B)** over time. Right: Quantification of Rac activity as AUC (mean ± SEM) during the first 125 s of cell spreading; dots represent individual cells. The numbers of cells tested for ICAM1 and pRGD were 54/22 for wild type, 43/50 for Tiam1^–/–^, 40/46 for Dock2^–/–^, 27/14 for Prex1^–/–^ Vav1^–/–^, respectively. Statistics are one-way ANOVA with Tukey’s multiple comparison corrections.

We proceeded to investigate Rac activity during adhesion and spreading. First, we used live-cell ratiometric TIRF-FRET imaging to investigate Rac activity during the initial attachment of neutrophils to either ICAM1 or pRGD surfaces in the presence of fMLP, from the moment the cells first touched the substrate. ICAM1 is a ligand specifically for β2-integrins (52), whereas pRGD is a promiscuous ligand for numerous integrins (53, 54). Prex1/Vav1 were required for the initial spreading of neutrophils on both ICAM1 and pRGD, whereas Tiam1 and Dock2 were dispensable (**Figures 2A, B**). In contrast, Rac activity at the basal cell surface was reduced in Rac-FRET Dock2^–/–^ neutrophils, but normal in Prex1/Vav1- and Tiam1-deficient cells (**Figures 2A, C**). Rac activity was highest around the periphery of spreading neutrophils in all genotypes, although this was difficult to discern in Rac-FRET Prex1^–/–^ Vav1^–/–^ cells due to their limited spreading (**Figure 2A**). Hence, there was no obvious correlation between the amount or distribution of Rac activity generated at the basal cell surface and the initial phase of neutrophil adhesion and spreading.

Next, we used live-cell ratiometric TIRF-FRET imaging to investigate Rac activity during the firm adhesion of neutrophils to ICAM1. Under the condition shown in **Figure 1**, where all GEFs were required for the firm adhesion to ICAM1 in the presence of fMLP, Rac activity was normal throughout, showing a lack of correlation between fMLP-stimulated adhesion and Rac activity (**Supplementary Figure 2**). Rac activity was reduced in Rac-FRET Prex1^–/–^ Vav1^–/–^ neutrophils plated in the absence of fMLP, and in Rac-FRET Dock2^–/–^ cells upon short-term exposure to fMLP (**Supplementary Figure 2**), suggesting that Prex1/Vav1- and Dock2-mediated Rac activity may be required prior to or during integrin activation. Even so, there was also no obvious correlation between neutrophil adhesion and the amount of Rac activity generated, as adhesion was also impaired in Rac-FRET Tiam1^–/–^ cells, although their Rac activity was normal with or without fMLP-stimulation.

Like fMLP, the chemoattractant KC (the mouse equivalent of human IL8), is another important GPCR ligand in neutrophil integrin activation (51). Therefore, we also tested the early adhesion and spreading of neutrophils to ICAM1 upon KC stimulation. Under these conditions, Rac activity was reduced in both Rac-FRET Dock2^–/–^ and Prex1^–/–^ Vav1^–/–^ neutrophils, although adhesion was impaired and spreading increased in Dock2^–/–^ but not Prex1^–/–^ Vav1^–/–^ cells (**Supplementary Figure 3A**). Finally, we used another approach to stimulate β2-integrin dependent neutrophil adhesion, by plating the cells on anti-CD18 antibody M18.2, which activates β2 integrins (55, 56). Under these conditions, Rac activity was reduced in Rac-FRET Dock2^–/–^ but not Prex1^–/–^ Vav1^–/–^ neutrophils, adhesion was reduced in Dock2^–/–^ and increased in Prex1^–/–^ Vav1^–/–^ cells and spreading was normal in Dock2^–/–^ and reduced in Prex1^–/–^ Vav1^–/–^ cells (**Supplementary Figure 3B**). Hence, again there was little correlation between Rac activity, adhesion and spreading, except that Rac activity and adhesion were both reduced in Dock2^–/–^ cells upon KC stimulation.

Overall, our data show that it is highly context-dependent which Rac-GEF generates Rac activity at the basal surface of adhering neutrophils, but there is no obvious correlation between this Rac activity and the ability of neutrophils to adhere and spread. It is possible that Rac activation prior to adhesion dictates which neutrophils adhere, but once the cells are adherent, Rac activity appears to be more important for other cell responses.

### Dock2-mediated Rac activity at the basal cell surface correlates with the ability of neutrophils to polarise and undergo random migration

**Figure 3:**
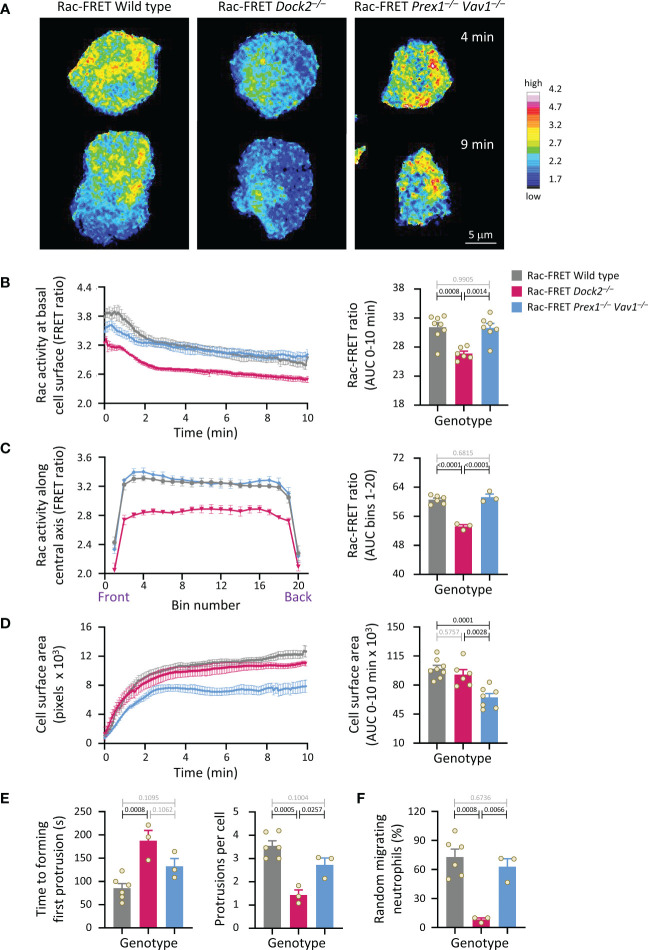
Dock2-mediated Rac activity correlates with the ability of neutrophils to polarise and undergo random migration. Neutrophils from wild type Rac-FRET mice (grey bars) or Rac-FRET mice deficient in the Rac-GEFs Dock2 (pink) or Prex1/Vav1 (blue) were plated onto glass coverslips at 37°C and imaged by live-cell TIRF-FRET microscopy for 10 min from the first point of contact with the slide, with frames taken every 5 s. Rac activity, cell spreading, polarisation and random migration were quantified. **(A)** Representative stills of **Supplementary Movie 1** from one experiment, at the 4 min and 9 min time points, as indicated. The pseudo-colour scale depicts high Rac activity (FRET ratio) in white/red and low Rac activity in blue. **(B)** Left-hand panel: Rac activity (FRET ratio; mean ± SEM) at the basal cell surface over time. Data are mean ± SEM of cells pooled from 3 independent experiments, with between 7 and 24 cells tested per genotype and experiment. Cells of different genotypes were compared directly in each experiment. Right: Quantification of Rac activity (AUC; mean ± SEM). **(C)** Front/back distribution of Rac activity. Left: line scans of Rac activity through the central longitudinal axis of the neutrophils in **(B)**, were made for each time point once the cells were spread, and a mean FRET ratio was calculated for each cell. These FRET ratios (mean ± SEM) grouped into 20 bins along the length of the cell. Right: Quantification of Rac activity along the longitudinal axis (mean ± SEM). **(D)** Spreading. Left: the basal cell surface area (mean ± SEM) of the cells in **(B, C)** over time. Right: quantification of spreading (AUC; mean ± SEM). **(E)** Quantification of the time until cells start polarising by forming a first leading edge protrusion (left) and the number of leading edge protrusions per cell over the 10 min observation period (right). **(F)** Quantification of the proportion of neutrophils undergoing random migration, defined as displacement by at least one cell diameter over the 10 min observation period. Data in **(E, F)** are mean ± SEM of the 3 independent experiments in **(B-D)**; each dot represents the mean of one experiment. Statistics in **(B-F)** are one-way ANOVA with Tukey’s multiple comparison corrections.

We investigated if Rac activity correlates with neutrophil polarisation and random migration. Rac-FRET Prex1^–/–^ Vav1^–/–^ and Dock2^–/–^ neutrophils were plated onto glass and imaged by live-cell TIRF-FRET microscopy for 10 min, from the first point of contact with the slide (**Figure 3A** and **Supplementary Movie 1**). Rac activity, spreading, polarisation and random migration were quantified. Mean Rac activity at the basal cell surface was reduced in Dock2^–/–^ cells throughout this time (**Figures 3A, B**). We investigated the spatial distribution of Rac activity in more detail over the central longitudinal axis of the cell. Rac activity was lower throughout the Rac-FRET Dock2^–/–^ neutrophil, whereas it was normal in Prex1^–/–^ Vav1^–/–^ cells (**Figure 3C**). Yet, as seen before, spreading was reduced in Rac-FRET Prex1^–/–^ Vav1^–/–^ rather than Dock2^–/–^ neutrophils, showing again the lack of correlation (**Figure 3D**). In contrast, Rac activity did correlate with the ability of neutrophils to polarise. Wild type Rac-FRET neutrophils polarised fully within the observation period, and Rac-FRET Prex1^–/–^ Vav1^–/–^ cells formed some, although often rudimentary protrusions, but Dock2^–/–^ cells failed to polarise (**Figure 3A** and **Supplementary Movie 1**). For both wild type and Prex1^–/–^ Vav1^–/–^ cells, the leading edge protrusion was formed in areas of highest Rac activity at the cell periphery (**Supplementary Movie 1**). We quantified the time it took for cells to start polarising by forming their first leading edge protrusion, and the number of leading edge protrusions formed throughout the observation period. By these measures, Rac-FRET Dock2^–/–^ cells showed impaired polarisation, whereas Prex1^–/–^ Vav1^–/–^cells did not (**Figure 3E**). We also quantified the proportion of cells that underwent random (unstimulated) migration, which we defined as a displacement of at least one cell diameter within the 10 min observation. Rac-FRET Dock2^–/–^ neutrophils showed a striking impairment in random migration (**Figure 3F**). Therefore, the reduced Rac activity in Rac-FRET Dock2^–/–^ neutrophils correlates with the ability of these cells to polarise and random migrate.

### Dock2-mediated Rac activity at the basal cell surface correlates with the ability of neutrophils to undergo chemokinesis

**Figure 4:**
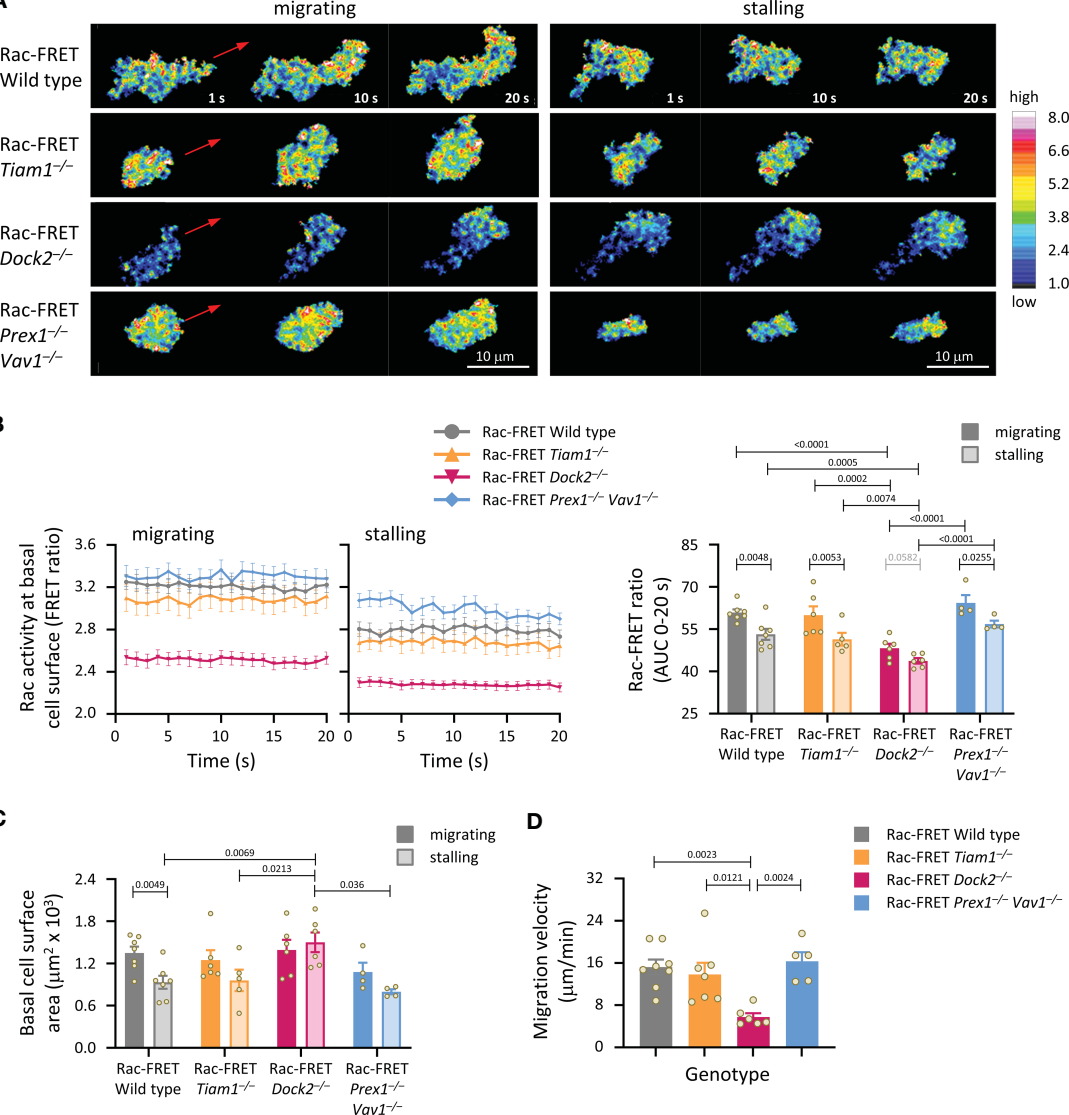
Dock2-mediated Rac activity correlates with migration velocity and the ability of neutrophils to contract during stalling phases of chemokinesis. Neutrophils from Rac-FRET mice (wild type; grey symbols) or Rac-FRET mice deficient in Tiam1 (yellow), Dock2 (pink), or Prex1/Vav1 (blue) were primed with 50 ng/ml GM-CSF and 20 ng/ml TNFα, plated onto ICAM1-coated ibidi slides at 37°C in the presence of 0.75 µM fMLP, and allowed to adhere for 5 min. Migrating cells were chosen for imaging by live-cell ratiometric TIRF-FRET microscopy for 2 min, at a frame interval of 1 s. Migrating phases and stalling phases of migration were defined as periods of at least 20 s duration where the cell either did or didn’t displace their centre of mass by 2 μm. **(A)** Stills of representative movies from one experiment, showing both active and stalling phases of chemokinesis for the same cell. The pseudo-colour scale depicts high Rac activity (high FRET ratio) in white/red and low Rac activity in blue. The red arrows show the direction of chemokinesis during migrating phases. **(B)** Left-hand panels: Rac activity (FRET ratio; mean ± SEM) at the basal cell surface during 20 s migrating and stalling phases of chemokinesis. Data are from 32 wild type, 18 Tiam1^–/–^, 18 Dock2^–/–^, and 8 Prex1^–/–^ Vav1^–/–^ cells, pooled from 4-7 independent experiments. Cells of different genotypes were compared directly in each experiment. Right: Quantification of Rac activity (AUC, mean ± SEM) during migrating and stalling phases of chemokinesis, from data on the left; each dot represents the mean of one experiment. **(C)** Spreading: basal surface area (mean ± SEM) of the cells in **(B)** integrated over the same 20 s migrating and stalling phases of chemokinesis. Statistics in **(B, C)** are two-way ANOVA with Sidak’s multiple comparisons test. **(D)** Velocity of chemokinesis. Data are mean ± SEM of 5-8 experiments; each dot represents the mean of one experiment. Statistics are one-way ANOVA with Tukey’s multiple comparisons test.

We analysed Rac activity during neutrophil chemokinesis (migration in a bath of chemoattractant). Neutrophils from wild type Rac-FRET mice and the various Rac-GEF deficient Rac-FRET mice were primed and allowed to adhere for 5 min to ICAM1 in a bath of fMLP. Migrating cells were imaged by live-cell TIRF-FRET microscopy and analysed during both active and stalling phases of migration, which we defined as periods of at least 20 s duration where the cell did or didn’t displace their centre of mass. In all genotypes except Dock2^–/–^, Rac activity was higher during migrating than stalling phases of chemokinesis (**Figures 4A, B**). Rac activity was reduced in Rac-FRET Dock2^–/–^ neutrophils both during migrating and stalling phases, but normal in Tiam1^–/–^ and Prex1^–/–^ Vav1^–/–^ cells (**Figures 4A, B**). Neutrophils from all genotypes except Rac-FRET Dock2^–/–^ contracted during stalling, resembling an amoeboid manner of migration (57). In contrast, Rac-FRET Dock2^–/–^ neutrophils failed to contract during stalling phases of chemokinesis (**Figure 4C**). This correlated with a strikingly lower migration velocity during chemokinesis (**Figure 4D**). Similarly, the velocity of chemokinesis was also reduced in Dock2^–/–^ neutrophils plated on ICAM1 or on activating anti-CD18 antibody in baths of KC (**Supplementary Figure 3**). Hence, Dock2 is required for the speed of neutrophil chemokinesis and for neutrophil contraction during stalling phases of chemokinesis, correlated with the amount of Rac activity generated at the basal cell surface.

### Dock2-mediated Rac activity at the basal cell surface correlates with the ability of neutrophils to migrate, but is not required for directional sensing

**Figure 5:**
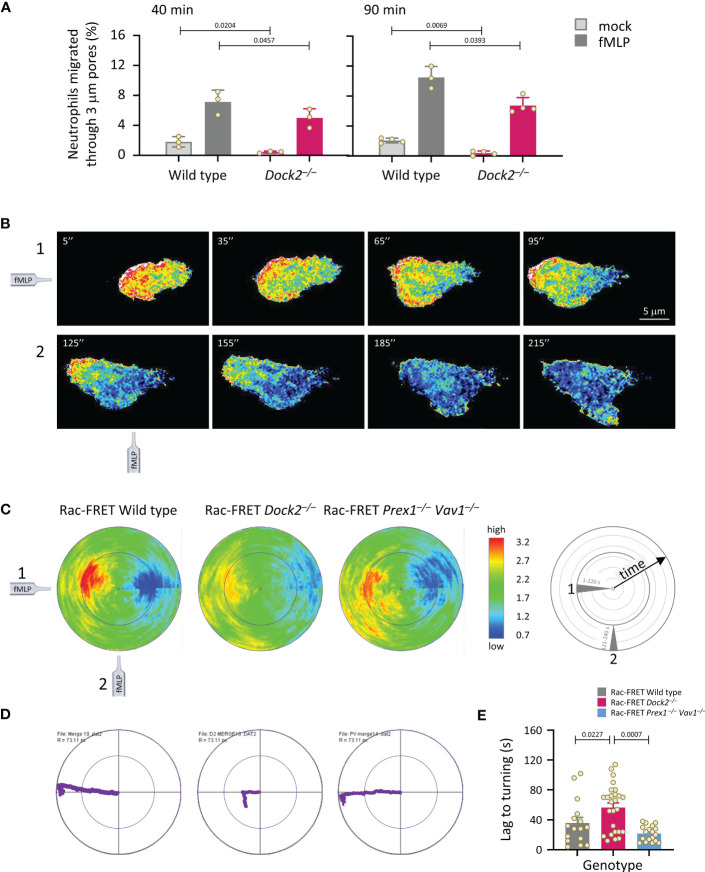
Dock2 is required for neutrophil migration, but not for directional sensing, although it dictates the speed of turning. **(A)** Transwell chemotaxis. Bone marrow cells from wild type (grey) and Dock2^–/–^ mice (pink) were subjected to transwell migration assays through 3 μm-pore polycarbonate filters in the presence (dark bars) or absence (light bars) of 3 μM fMLP, for 40 or 90 min at 37°C. Transmigrated neutrophils were identified and enumerated by flow cytometry. Data are mean ± SEM of 3-4 independent experiments; each dot represents the mean of one experiment. Statistics are two-way ANOVA with Sidak’s multiple comparison corrections. **(B-E)** Micropipette chemotaxis assays. Neutrophils from wild type and GEF-deficient Rac-FRET mice, as indicated, were allowed to attach to glass coverslips at 37°C and stimulated with a point source of 1 μM fMLP delivered from a microinjection needle placed ~20 μm ‘west’ of the cell (position 1), while Rac activity was imaged by live-cell ratiometric TIRF-FRET imaging for 4 min, at a frame interval of 2 s. Half-way through the experiment, the needle was moved 90˚ (‘south’, position 2) to measure the ability of the cell to turn. **(B)** Stills from a representative movie of a wild type Rac-FRET neutrophil migrating towards the fMLP needle in positions 1 (west, 1-120 s) and 2 (south, 121-240 s), as indicated. **(C)** Polar plots of micropipette experiments as in **(B)**, quantifying the intensity and distribution of Rac activity around the periphery of the migrating cell over time, with the first frame at the centre of the circle and the last at the plot edge. The fMLP micropipette was positioned first west and then south, as indicated in the schematic. The pseudo-colouring in **(B, C)** depicts high Rac activity (FRET ratio) in red and low Rac activity in blue. Plots from all cells per genotype from 1 experiment representative of 4 were combined, to quantify mean Rac activity along the cell periphery over time. The numbers of neutrophils in the plots shown are 11 wild type, 10 Dock2^–/–^, and 13 Prex1^–/–^ Vav1^–/–^ Rac-FRET cells. **(D)** Movement plots, depicting the mean path migrated by the cells directly above. **(E)** The time it took for neutrophils to form a new leading edge after the micropipette was moved to position 2 is quantified. Data are mean ± SEM of 16 wild type, 26 Dock2^–/–^, and 18 Prex1^–/–^ Vav1^–/–^ Rac-FRET cells pooled from 3 independent experiments; each dot represents the mean of one cell. Statistics are one-way ANOVA with Tukey’s multiple comparison corrections.

Next, we analysed neutrophil chemotaxis (directional migration in a gradient of chemoattractant). Initially, neutrophils from wild type and Dock2^–/–^ Rac-FRET mice were subjected to transwell chemotaxis assays. fMLP stimulation induced robust chemotaxis in wild type neutrophils, but less in Dock2^–/–^ cells at both time points tested, 40 and 90 min (**Figure 5A**). In addition, random migration, in the absence of fMLP, was again lower in the Rac-FRET Dock2^–/–^ neutrophils (**Figure 5A**). For comparison, we previously showed that fMLP-stimulated chemotaxis is reduced in Prex1^–/–^Vav1^–/–^ neutrophils under the same conditions, whereas their random migration was normal (21), and ibidi chemotaxis assays showed that the impaired chemotaxis of Prex1^–/–^Vav1^–/–^ neutrophils is due to reduced velocity rather than reduced directional sensing (25). Next, we performed micropipette chemotaxis assays combined with live-cell ratiometric TIRF-FRET imaging of Rac activity in wild type, Dock2^–/–^ and Prex1^–/–^ Vav1^–/–^ Rac-FRET neutrophils. We measured directional sensing and the ability to turn in a changing chemoattractant gradient, to correlate these responses with Rac activity. Neutrophils from wild type and GEF deficient Rac-FRET mice were stimulated with a point source of fMLP over 4 min, the micropipette being placed first ‘west’ of the cell, and then ‘south’ half-way through the experiment (**Supplementary Movie 2**). Cells of all genotypes were able to orient themselves within the gradient and turn when the chemoattractant source was moved, although Rac-FRET Dock2^–/–^ cells migrated less far (**Figures 5B–D**). In all genotypes, Rac activity was highest at the cell front that faced the chemoattractant source, decreased when the chemoattractant was removed, and increased again at the new leading edge which formed towards the new source. Rac activity was low throughout in Rac-FRET Dock2^–/–^ cells, correlated with the lesser distance migrated, but the repositioning of that low Rac activity was preserved, correlated with the ability of Dock2^–/–^ cells to sense direction and turn (**Figures 5B–D**). However, while it took wild type cells 36 s to form a new leading edge for turning, this took 57 s in Dock2^–/–^ neutrophils (**Figure 5E**). Hence, Dock2 controls the amount but not the spatiotemporal distribution of Rac activity at the cell periphery, and is required for random neutrophil migration and chemokinesis but not for chemotaxis, although it does control the speed of turning.

### Dock2 generates Rac activity at the leading edge of migrating neutrophils

**Figure 6:**
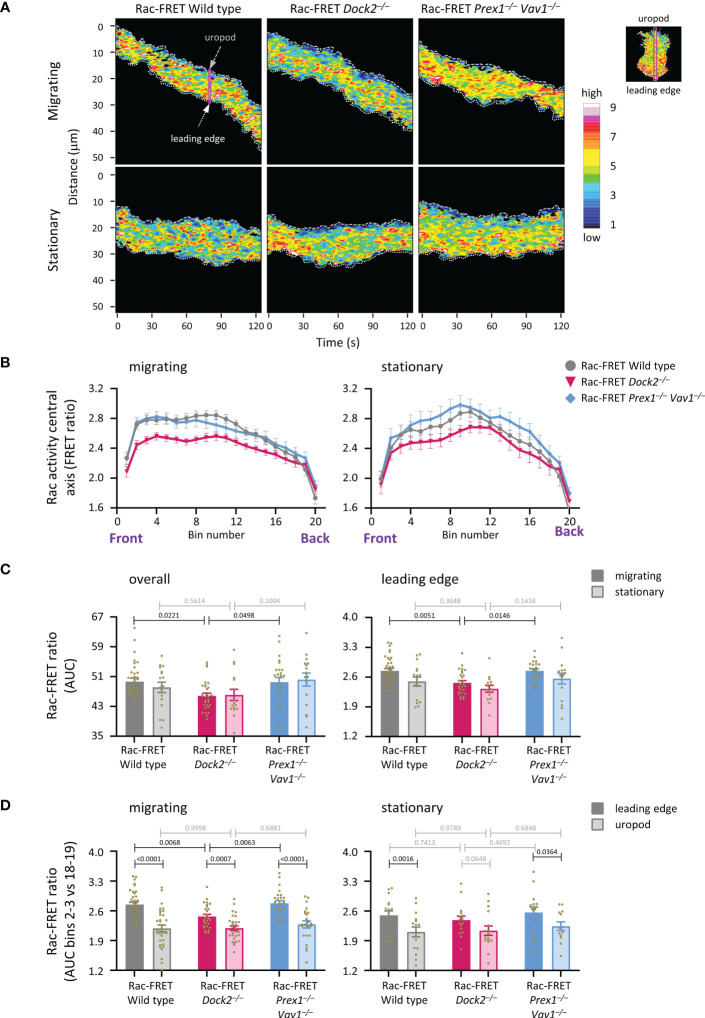
Dock2 generates Rac activity at the leading edge during neutrophil chemotaxis. Neutrophils from wild type Rac-FRET mice (grey) or Rac-FRET mice deficient in Dock2 (pink) or Prex1/Vav1 (blue) were tested by micropipette chemotaxis assay as in **Figures 5B–E**, except that the position of the fMLP micropipette needle was kept constant. Live-cell ratiometric TIRF-FRET imaging was performed for 2 min, at a frame interval of 1 s. Line scans through the central longitudinal axis of the cell were prepared for each frame to compare the height and localisation of Rac activity between actively migrating and stationary cells, as indicated. **(A)** Representative kymographs plotting Rac activity (FRET ratio) along the central longitudinal axis of the cell as a function of time. The leading edge is indicated by the white dotted line, the uropod by the grey stippled line. The pink box shows an example of one timepoint. The fMLP micropipette was positioned south of the cells shown. **(B)** Rac activity (FRET ratio) along the central axis from the leading edge to the uropod of cells in an fMLP gradient over time, comparing migrating and stationary cells. Left-hand panel: Rac activity throughout the length of the cell, AUC of bins 1-20. Right-hand panel: Rac activity at the leading edge, AUC of bins 2-3. Data are mean ± SEM of cells pooled from 4 independent experiments. The numbers of migrating and stationary neutrophils in the plots shown are 35/17 wild type, 29/16 Dock2^–/–^, and 25/16 Prex1^–/–^ Vav1^–/–^, respectively. **(C)** Quantification of overall Rac activity in migrating and stationary cells from **(B)**, as AUC (mean ± SEM). **(D)** Quantification of Rac activity at the leading edge (bins 2 and 3) and uropod (bins 18 and 19) of the migrating and stationary cells in **(A)**, expressed as AUC (mean ± SEM). Statistics in **(C, D)** are two-way ANOVA with Sidak’s multiple comparisons test.

To understand better the role of Dock2 in generating Rac activity during neutrophil migration, we compared the amount and localisation of Rac activity between actively migrating and stationary wild type and GEF-deficient Rac-FRET cells by micropipette chemotaxis assay (with a constant position of the fMLP needle). We defined migrating cells as those that translocated at least half their body length during the 2 min observation period. Live-cell TIRF-FRET imaging was performed and Rac activity along the central longitudinal axis of the cell plotted as a function of time in kymographs (**Figure 6A**). Peak Rac activity was higher at the leading edge than the uropod in migrating neutrophils, but shifted away from the leading edge towards the centre in stationary cells, as we previously described (41). This shift was seen in all genotypes (**Figure 6B**). Peak Rac activity was lower in migrating Rac-FRET Dock2^–/–^ neutrophils than wild type or Prex1^–/–^ Vav1^–/–^ cells, both overall and at the leading edge in particular (**Figures 6B, C**). Rac activity was higher at the leading edge than the uropod in all genotypes, except in stationary Rac-FRET Dock2^–/–^ cells, and was lost from the leading edge of migrating Rac-FRET Dock2^–/–^ cells (**Figure 6D**). Hence, Dock2 is required for generating Rac activity at the leading edge of migrating neutrophils.

### Dock2 stabilises Rac activity at the uropod of migrating neutrophils

**Figure 7:**
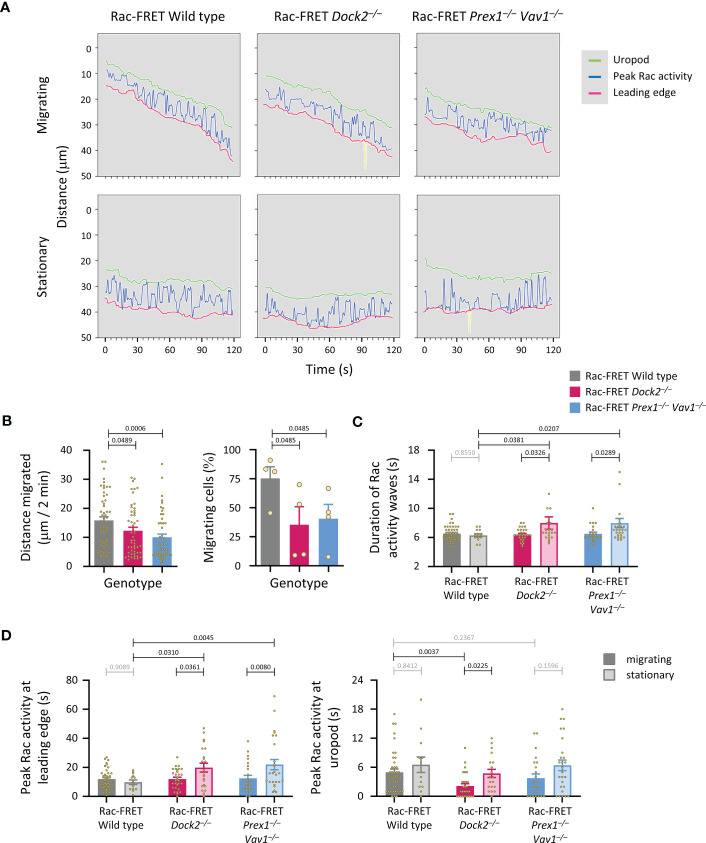
Dock2 stabilises Rac activity at the uropod during neutrophil chemotaxis. Neutrophils from wild type Rac-FRET mice (grey) or Rac-FRET mice deficient in Dock2 (pink) or Prex1/Vav1 (blue) were tested by micropipette chemotaxis assay as in **Figure 6**. Live-cell ratiometric TIRF-FRET imaging was performed for 2 min, at a frame interval of 1 s. The height and localisation of peak Rac activity (FRET ratio) along line scans through the central longitudinal axis of the cell was determined for each frame in actively migrating and stationary cells, as indicated. **(A)** Representative traces from one experiment, showing the position of peak Rac activity (blue) within migrating and stationary cells in an fMLP gradient over time, in relation to the leading edge (pink) and uropod (green). The traces are from the same cells as in **Figure 6A**. **(B)** Distance migrated and proportion of cells that migrated at least half their body length during the 2 min observation period. Data are mean ± SEM of 56 wild type, 52 Dock2^–/–^, and 51 Prex1^–/–^ Vav1^–/–^ neutrophils pooled from 4 independent experiments that directly compared the different genotypes. Statistics are one-way ANOVA with Tukey’s multiple comparisons test. **(C)** Duration (mean ± SEM) of peak Rac activity waves between leading edge or uropod of the cells in **(B)**, comparing migrating and stationary cells. **(D)** Time of peak Rac activity localisation within 0.8 μm of the leading edge (left-hand panel) or uropod (right-hand panel) over the 2 min observation period, comparing the same migrating and stationary cells as in **(B)**. Statistics in **(C, D)** are two-way ANOVA with Sidak’s multiple comparisons test.

We previously established that peak Rac activity oscillates between the leading edge and uropod of migrating neutrophils, with a duration of ~8 s per wave (41). Here, we investigated if Rac-GEFs generate these waves, by determining the localisation of peak Rac activity along the central longitudinal cell axis of Rac-FRET Prex1^–/–^ Vav1^–/–^ and Dock2^–/–^ neutrophils over 2 min (**Figure 7A**). Neutrophils from both GEF-deficient strains migrated less far, and a smaller proportion migrated at all (**Figure 7B**). Waves of Rac activity were observed both in migrating and stationary cells. In migrating cells, these waves had a duration of 6.5 ± 0.2 s, slightly less than we observed previously, regardless of genotype. In stationary cells, the waves were similar in wild type (6.3 ± 0.2 s) but slowed to 8.0 ± 0.8 and 8.0 ± 0.6 s in Rac-FRET Dock2^–/–^ and Prex1^–/–^ Vav1^–/–^, respectively (**Figure 7C**). Moreover, peak Rac activity dwelled longer at the leading edge in stationary cells of both GEF-deficient genotypes than wild type (**Figure 7D**). Hence, both Dock2 and Prex1/Vav1 regulate the front/back polarity of Rac activity, correlated with a reduced distance migrated and lesser likelihood of migrating overall. Additionally, Rac activity was lost from the uropod of migrating Rac-FRET Dock2^–/–^ cells. Therefore, Dock2 is also required for stabilising Rac activity at the uropod of migrating neutrophils, which may contribute to the control of migration speed by this GEF.

### Dock2-mediated Rac activity correlates with the speed of particle engulfment during phagocytosis

**Figure 8:**
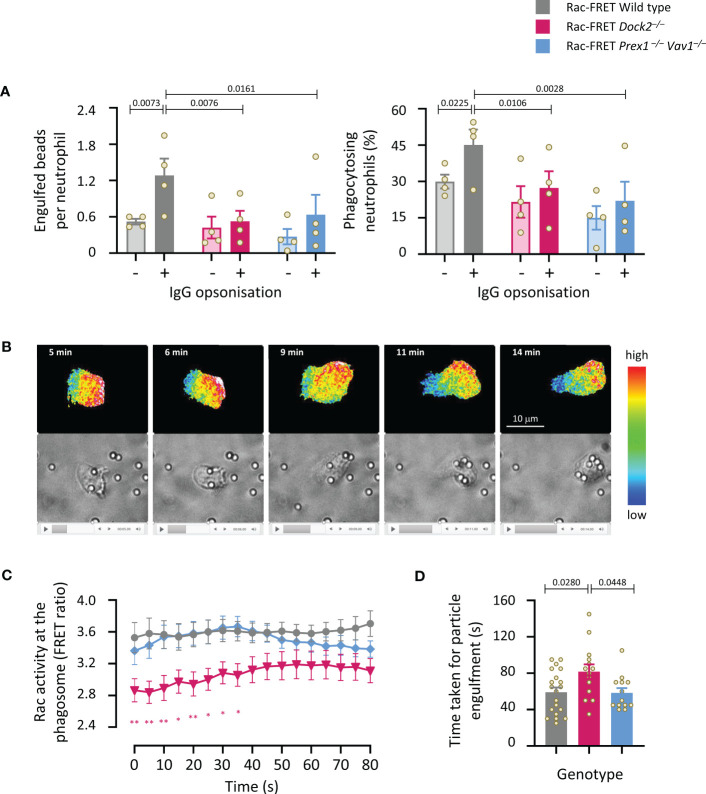
Dock2-mediated Rac activity correlates with the speed of particle engulfment during the phagocytosis of IgG-coated particles. **(A)** Phagocytosis (fixed cells): Purified neutrophils from wild type Rac-FRET mice (grey) or Rac-FRET mice deficient in Dock2 (pink) or Prex1/Vav1 (blue) were primed with 20 ng/ml TNFα and 50 ng/ml GM-CSF for 45 min at 37°C, and then incubated for 15 min at 37°C with 2 µm Fluoresbrite^®^ yellow/green carboxylate microspheres that had or had not been opsonised with IgG, as indicated, at a ratio of 10 particles/cell. Cells were fixed, stained with FITC-Gr1 antibody, and imaged by widefield fluorescence microscopy. Images were analysed by Fiji for the number of particles taken up per cell and the percentage of neutrophils containing at least one particle. Data are mean ± SEM of 4 independent experiments. Statistics are two-way ANOVA with Sidak’s multiple comparisons test. **(B-D)** Phagocytosis (live cells): Neutrophils exposed to IgG-opsonised beads as in **(A)** were live-imaged by ratiometric TIRF-FRET microscopy for 15 min, with frames taken every 5 s, and with brightfield imaging in parallel to visualise Rac activity, cells and particles at the same time. **(B)** Stills from **Supplementary Movie 3** of a wild type Rac-FRET neutrophil chasing IgG-coated particles. Top panels: Rac activity (FRET ratio). Bottom: brightfield. **(C)** Quantification of Rac activity (FRET ratio; mean ± SEM) around the forming phagosome over time. Data are mean ± SEM of 20 wild type, 14 Dock2^–/–^, and 13 Prex1^–/–^ Vav1^–/–^ cells pooled from 3 independent experiments. **(D)** Time taken for full engulfment of an IgG-opsonised particle. Data are mean ± SEM of the 3 experiments in **(C)**. Statistics in **(C, D)** are one-way ANOVA with Tukey’s multiple comparisons test. **(C)** * signifies p<0.05, ** means p<0.01.

We investigated the roles of Dock2 and Prex1/Vav1 in the phagocytosis of carboxylate beads with or without IgG opsonisation. The opsonisation, which stimulates Fc receptors, increased the number of beads taken up by wild type Rac-FRET cells, and the proportion of cells that phagocytosed any particles within 15 min (**Figure 8A**). Both Dock2- and Prex1/Vav1-deficient cells showed a reduction in the number of opsonised particles taken up and the proportion of cells that did take them up, showing that these GEFs are important for Fc receptor-dependent phagocytosis (**Figure 8A**). Live-cell ratiometric TIRF-FRET imaging showed that Rac activity was highest at the leading edge of neutrophils migrating towards the opsonised beads, particularly as the cells made first contact with the beads, and also at the forming phagosome (**Figure 8B** and **Supplementary Movie 3**). We quantified Rac activity around the phagosome, and the time it took for neutrophils to engulf each particle. Both were normal in Rac-FRET Prex1^–/–^ Vav1^–/–^ neutrophils but reduced in Dock2^–/–^ cells (**Figures 8C, D**). Hence, the Dock2-mediated Rac activity around the phagosome correlates with the speed with which neutrophils phagocytose opsonised particles.

### Dock2 is required for neutrophil recruitment during aseptic peritonitis

**Figure 9:**
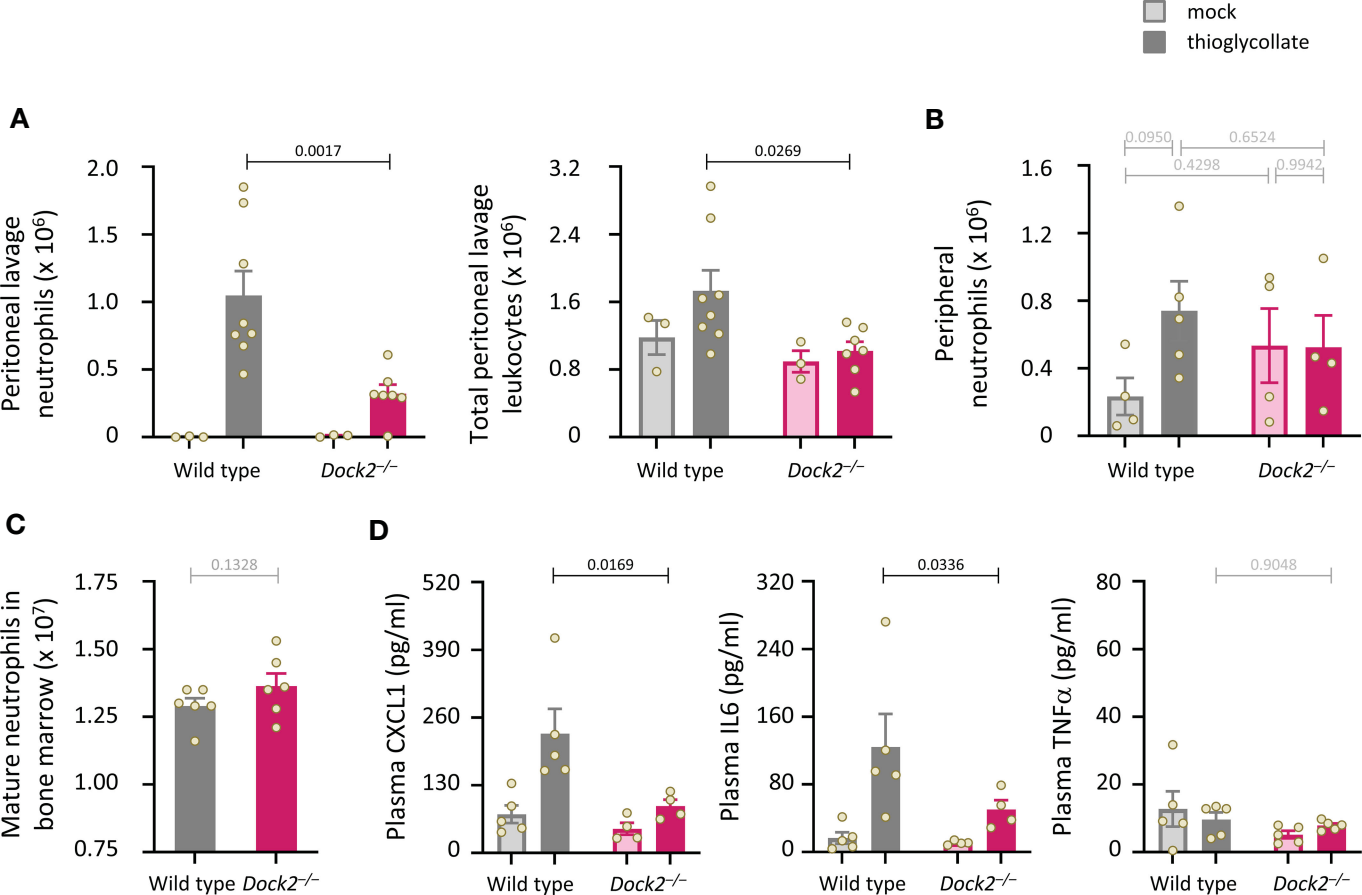
Dock2 is required for neutrophil recruitment during aseptic peritonitis. **(A)** Wild type (grey) and Dock2^–/–^ (pink) mice were treated with 0.25 ml sterile 3% thioglycollate (TGC, *i.p.*, dark bars), or were mock-treated (light bars), and culled 3 h later. Peritoneal lavages were performed, and lavage leukocytes were stained and analysed by flow cytometry. Neutrophils (left-hand panel) were identified by Cd11b^hi^, Gr1^hi^ staining and enumerated taking into account the lavage volume recovered, and were compared to the number of total peritoneal leukocytes (right-hand panel). Data are mean ± SEM of mice pooled from two independent cohorts, with 1-2 mock-treated and 3-4 TGC-treated animals per genotype in each experiment. Overall numbers were 3 mock-treated mice per genotype, and 8 wild type and 7 Dock2^–/–^ TGC-treated mice; each dot represents one mouse. Statistics are two-way ANOVA with Sidak’s multiple comparisons test. **(B)** Rac-FRET Wild type (grey) and Rac-FRET Dock2^–/–^ (pink) mice were treated with TGC or mock-treated as in **(A)**, culled 3 h later, peripheral blood collected and analysed by flow cytometry for the number of neutrophils. Data in **(B)** are mean ± SEM of mice pooled from two independent cohorts, with 2-3 animals per group in each experiment; each dot represents one mouse. **(C)** Numbers of mature neutrophils isolated from the bone marrow of Rac-FRET Wild type and Rac-FRET Dock2^–/–^mice. Data are mean ± SEM of mice from 6 independent experiments; each dot represents one mouse. Statistics are paired t-test. **(D)** The blood plasma of the mice in **(B)** was analysed by ELISA for levels of the indicated chemokines and cytokines. Statistics in **(B, D)** are two-way ANOVA with Sidak’s multiple comparisons test on square root-transformed data.

Finally, we subjected Dock2^–/–^ mice to intraperitoneal challenge with thioglycollate (TGC) to induce sterile peritonitis and assessed neutrophil recruitment after 3 h. Dock2^–/–^ mice recruited fewer neutrophils to the inflamed peritoneum than wild type mice, and this reduced recruitment accounted for an overall reduction in the number of peritoneal leukocytes (**Figure 9A**). Peripheral neutrophil numbers were comparable between Rac-FRET Wild type and Dock2^–/–^ mice and were not significantly affected by the TGC-treatment, suggesting the recruitment defect in Dock2-deficient mice was not a consequence of lower neutrophil numbers overall (**Figure 9B**). Accordingly, the numbers of mature Rac-FRET Dock2^–/–^ mice in the bone marrow were also normal (**Figure 9C**). Plasma levels of the chemokine CXCL1 and cytokine IL6 increased upon TGC treatment in Rac-FRET Wild type mice but were significantly lower in Dock2^–/–^ mice, whereas plasma TNFα was unaffected under these conditions and remained low throughout (**Figure 9D**). The reduced levels of CXCL1 may explain the impaired recruitment of Dock2-deficient neutrophils to the inflamed peritoneum. We conclude that Dock2 is an important regulator of neutrophil migration *in vivo* as well as *in vitro*.

## Discussion

In this study, we correlated patterns of Rac activity generated by the Rac-GEFs Dock2, Tiam1 and Prex1/Vav1 with the cell responses that these Rac-GEFs generate in adherent neutrophils. Our main finding is that Dock2, above the other Rac-GEFs, is an important regulator of neutrophil polarisation, migration and phagocytosis, and that this GEF produces characteristic patterns of Rac activity which correlate with its roles in these cell responses.

We used ratiometric confocal TIRF-FRET imaging in live cells throughout this study, which afforded greater resolution and signal-to-noise ratio than the widefield FRET imaging we employed previously in our study that first established the Rac-FRET reporter mouse (41). We had kept the expression level of the Raichu-Rac FRET reporter in this mouse strain deliberately low (1% over endogenous Rac in neutrophils), so as not to interfere with Rac-dependent neutrophil responses, which we confirmed (41), but this meant that the FRET signal strength generated by widefield imaging was limiting. The TIRF mode allowed us to concentrate on Rac activity at the basal cell surface which touches the substrate, where the cell forms new attachments between adhesion receptors and ligands during spreading, polarisation and migration, and where it maintains, reinforces and turns over these attachments during any adhesion-dependent process. Such attachments include integrin-dependent focal adhesions and others such as interactions between immunoglobulins and Fc receptors. Therefore, a focus on the basal cell surface was biologically relevant as well as having technical advantages. In addition, Rac activity was previously shown to be highest at the basal surface of endothelial cells, and particularly at forming basolateral protrusions (58). An obvious drawback of TIRF-FRET imaging is that we could not take into consideration Rac activity elsewhere in the cell, and of course we could only measure Rac activity in cells that were able to adhere.

We did not see correlations between the amount or spatiotemporal distribution of Rac activity at the basal cell surface and the ability of neutrophils to adhere and spread. All GEFs investigated contributed to adhesion, and Prex1/Vav1 were required for spreading. However, Rac activity was most reduced in Rac-FRET Dock2^–/–^ cells, although these cells spread more than wild type under some conditions. The importance of the various GEFs in adhesion and spreading was context dependent, differing between various integrin-ligand surfaces, and with the presence, identity and timing of chemoattractant used to activate integrins, but associations with Rac activity were not obvious under any condition. This could mean that Rac-GEF activity at the basal cell surface is not required for adhesion and spreading, whereas Rac activity elsewhere is, but this seems unlikely considering numerous studies in various cell types linking Rac activity to adhesion complexes (59). Another possibility is that Rac activity during adhesion and spreading is governed by other Rac-GEFs, but again this seems unlikely as the GEFs investigated here did control these cell responses. It is possible, of course, that we missed subtle spatiotemporal patterns of Rac activity which are important during adhesion and spreading. However, disconnects between Rac activity and spreading were seen previously in fibroblasts and hematopoietic stem cell derived neutrophil-like cells, so may reflect a true lack of dependence (60–62). Steffen et al. used Rac1^–/–^ mouse embryonic fibroblasts to show that Rac1 is essential for lamellipodia formation, membrane ruffling, random migration and hepatocyte growth factor-induced chemotaxis, whereas spreading was either normal or increased depending on the surface that the cells adhered to (62). Gu et al. showed that β2 integrin-dependent spreading is increased in Rac1^–/–^ neutrophils derived from mouse haematopoietic stem cells, whereas it is decreased in Rac2^–/–^ neutrophils (60). Similarly, Pestonjamasp et al. showed that neutrophils from mice with myeloid Rac1 deficiency spread more than wild type during chemotaxis due to a tail retraction defect, whereas Rac2^–/–^ neutrophils spread less (61). Altogether, we suppose that Rac activity prior to adhesion is important in determining which cells will be able to adhere, but once cell are adherent, Rac activity becomes more important for polarisation and migration.

Indeed, the size, timing and localisation of Rac activity did correlate with other neutrophil responses. Rac activity was low over the whole basal surface of Rac-FRET Dock2^–/–^ neutrophils, which had a reduced ability to polarise, form leading edge protrusions, undergo random migration, and perform chemokinesis. During chemokinesis, Rac activity was lower during stalling phases than during active migration in all genotypes, but it was lowest throughout in Rac-FRET Dock2^–/–^ neutrophils, which did not contract during stalling phases and showed reduced migration velocity. In contrast, Dock2 was not required for directional sensing in chemoattractant gradients, and is therefore dispensable for chemotaxis, confirming our previous report (32). The spatial localisation of Rac activity generated by Dock2 during chemotaxis was correct, but only a low amount of active Rac was made, so it appears that this overall reduction is responsible for the reduced migration speed. In fact, none of the GEFs we investigated here showed impairments in directional sensing, although Dock2 deficiency caused a delay in the formation of a new leading edge when the cell turned in a changing gradient. Rac1, rather than Rac2, is the small GTPase that confers directional migration (63), so likely a Rac1-GEF, which remains to be identified, controls the ability to sense direction. Vav family GEFs have a preference for Rac1 over Rac2, but no absolute specificity (21), and none of the Vav GEFs control directional sensing (9). We recently analysed the neutrophil proteome, which identified 39 different Rho-GEFs in addition to those studied here (46). Several of these Rho-GEFs, including Pix, Sos and Swap70, are able to activate Rac, but any substrate specificity for Rac1 over Rac2 remains to be evaluated. αPix was previously shown to regulate directional sensing during neutrophil chemotaxis, but this was attributed to its Cdc42-GEF rather than its Rac-GEF activity (64), so the GEF that generates Rac activity for directional sensing remains to be identified.

We investigated the spatiotemporal distribution of Rac activity during neutrophil migration in more detail, which showed that Dock2, rather than Prex1/Vav1, is required for generating Rac activity at the leading edge of migrating cells. Moreover, Dock2 was also required for Rac activity at the uropod. In contrast, both Dock2 and Prex1/Vav1 were important for the duration of peak Rac activity waves that traverse the cell, as well as for the localisation of Rac activity at the leading edge of stationary cells, consistent with a reduction of cell migration speed and likelihood of migrating overall. Furthermore, we compared the requirements for Dock2 and Prex1/Vav1 in Fc-receptor dependent phagocytosis. Both Dock2- and Prex1/Vav1-deficient cells engulfed fewer opsonised particles and were less likely to take up any opsonised particles. However, only Rac-FRET Dock2^–/–^ cells showed a lack of Rac activity at the phagosome, and this correlated with a reduction in the speed of particle engulfment. Finally, we showed that neutrophil recruitment is impaired in Dock2-deficient mice during sterile peritonitis, which showed that this GEF is an important regulator of neutrophil migration not only *in vitro* but also *in vivo*. Dock2 mice were previously shown to have reduced neutrophil recruitment in an infection model (34), but this is the first demonstration of its importance in sterile inflammation. It would be very interesting to investigate Rac activity in neutrophils under inflammatory conditions *in vivo*. We previously used intravital FLIM-FRET multiphoton microscopy to measure Rac activity in live Rac-FRET mice in cancer models (41), and we used RhoA-FRET LysM^Cre^ mice in a similar manner to measure RhoA activity during *S. aureus*-induced neutrophil swarming in the skin (65). Similar experiments could be done in the future with Dock2^–/–^mice expressing the Raichu-Rac FRET reporter specifically in neutrophils to measure the influence of Dock2 on Rac activity under inflammatory conditions *in vivo*.

Together, the data from mice suggest that lack of Dock2 activity reduces neutrophil recruitment during inflammatory and infectious diseases, as well as the neutrophil effector responses that clear pathogens. Hence, if one could inhibit DOCK2 in human patients with inflammatory disease, any associated neutrophil-mediated tissue injury would likely be reduced. However, resistance to infection would be impaired at the same time. Indeed, a point mutation resulting in loss of DOCK2 expression was found in four siblings with severe immunodeficiency, associated with impaired actin polymerisation, polarisation and ROS production in neutrophils (35). Furthermore, the importance of Dock2 in human immune disease is not restricted to neutrophils. We recently identified a risk allele associating decreased DOCK2 expression in patients with severe COVID-19, associated with changes in monocytes, macrophages and dendritic cells (36). Moreover, six unrelated patients with severe immunodeficiency caused by loss-of-function mutations in DOCK2 showed impaired responses in T cells, B cells, and NK cells (66, 67).

We considered why Dock2 would generate more Rac activity in adhering neutrophils than the other GEFs. One obvious difference is that Dock2 is a DOCK-type GEF with a DHR2 catalytic domain, whereas the others are Dbl-type, with a catalytic DH domain (9). However, their mechanism of catalysis is the same, so this is unlikely to be an explanation. Rather, the signalling mechanisms that regulate Dock2 are somewhat different to those that control Prex1, Vav1 and Tiam1, although there is substantial overlap. Dock2 is activated by RhoG binding to the Dock-adaptor protein Elmo, and by recruitment to the plasma membrane through the signalling lipids PIP_3_ and phosphatidic acid, and the GEF activates Rac in response to the stimulation of GPCRs (32, 33). In contrast, P-Rex1 is activated by PIP_3_ and by the Gβγ subunits of heterotrimeric G proteins, and signals in response to the activation of GPCRs, E-selectin and toll-like receptor 4 (15, 68, 69). Vav GEFs are activated by tyrosine phosphorylation and signal downstream of various receptors, including GPCRs, integrins, and Fc receptors (16). Tiam1 is activated by Ras and modulated by multiple other mechanisms (17), and we have shown recently that it paradoxically limits the activation of Rac1 and Rac2 upon fMLP stimulation of neutrophils adhering to ICAM1 (manuscript submitted). Whether any of these differences in upstream regulators dictate the spatiotemporal patterns of Rac activity through which Dock2 controls neutrophil polarisation and migration remains to be seen. An alternative would be that there is simply more Dock2 protein than other Rac-GEFs in mouse neutrophils, but our recent proteomic analysis does not support this notion (46). Or Dock2 may have a different subcellular localisation to the other GEFs. All GEFs we investigated, with the possible exception of Tiam1, are cytosolic in basal neutrophils and translocate to the plasma membrane upon neutrophil activation (9), but there may be subtle differences that have been overlooked. Finally, GEFs can form complexes not just with their upstream regulators and Rac, but also with the effector proteins activated by Rac. Differential binding to effector proteins is, for example, known to govern different roles of Prex1 and Tiam1 in the migration of fibroblasts (70). It is possible that Dock2 binds to a different set of Rac effectors than the Dbl-type GEFs. It would be interesting to evaluate this possibility in the future.

The complexity of Rac-dependent responses necessitates the tight control mechanisms described here to ensure that neutrophils provide robust immunity without causing inflammatory disease. Rac-GEFs are key elements of these tight control mechanisms, and our study confirms that they each regulate specific aspects of neutrophil adhesion and migration. Among these GEFs, Dock2 stands out as a key regulator of polarity and of the speed of migration and phagocytosis, correlated with the patterns of Rac activity generated by this GEF.

## Data availability statement

The original contributions presented in the study are included in the article/**Supplementary Material**. Further inquiries can be directed to the corresponding author.

## Ethics statement

The animal study was reviewed and approved by Babraham Animal Welfare Ethical Review Body.

## Author contributions

PAM, A-KEJ, EJM, CP, SAC and JYC planned, conducted and analysed the experiments and made graphs. PAM and A-KEJ wrote part of the manuscript. SW planned and helped with FRET imaging and image analysis. HO developed image analysis macros and helped with image analysis. AS-P supervised experimental design and statistical analysis. AM provided the Tiam1-deficient mouse strain and advice on Tiam1 signalling. YF provided the Dock2-deficient mouse strain and advice on Dock2 signalling. HCEW planned and supervised the project, obtained the funding and wrote the manuscript. All authors reviewed the manuscript.
