# Supplementary material for: Dock2 generates characteristic spatiotemporal patterns of Rac activity to regulate neutrophil polarisation, migration and phagocytosis
Source: Front Immunol. 2023 Jun 13;14:1180886. doi: 10.3389/fimmu.2023.1180886 (PMC10293741; doi:10.3389/fimmu.2023.1180886)
Supplement: Supplementary file 1 [file DataSheet_1.pdf]

Supplementary Material

Dock2 generates characteristic spatiotemporal patterns of Rac activity to regulate neutrophil polarisation, migration and phagocytosis

Polly A. Machin [1,2], Anna-Karin E. Johnsson [1,2], Ellie J. Massey [2], Chiara Pantarelli [2], Stephen A. Chetwynd [2], Julia Y. Chu [2], Hanneke Okkenhaug [3], Anne Segonds-Pichon [4], Simon Walker [3], Angeliki Malliri [5], Yoshinori Fukui [6], Heidi C. E. Welch [2,*]

[1] These authors contributed equally to this work and share first authorship.

[2] Signalling Programme, [3] Imaging Facility and [4]Bioinformatics Facility, The Babraham Institute, Babraham Research Campus, Cambridge, United Kingdom

[5] Cell Signalling, Cancer Research UK Manchester Institute, Alderly Park, United Kingdom

[6] Division of Immunogenetics, Department of Immunobiology and Neuroscience, Medical Institute of Bioregulation, Kyushu University, Fukuoka, Japan

*** Correspondence:**
Heidi Welch
heidi.welch@babraham.ac.uk

Content:

Supplementary Figures 1-3 and Legends

Legends to Supplementary Movies 1-3

Supplementary Figures

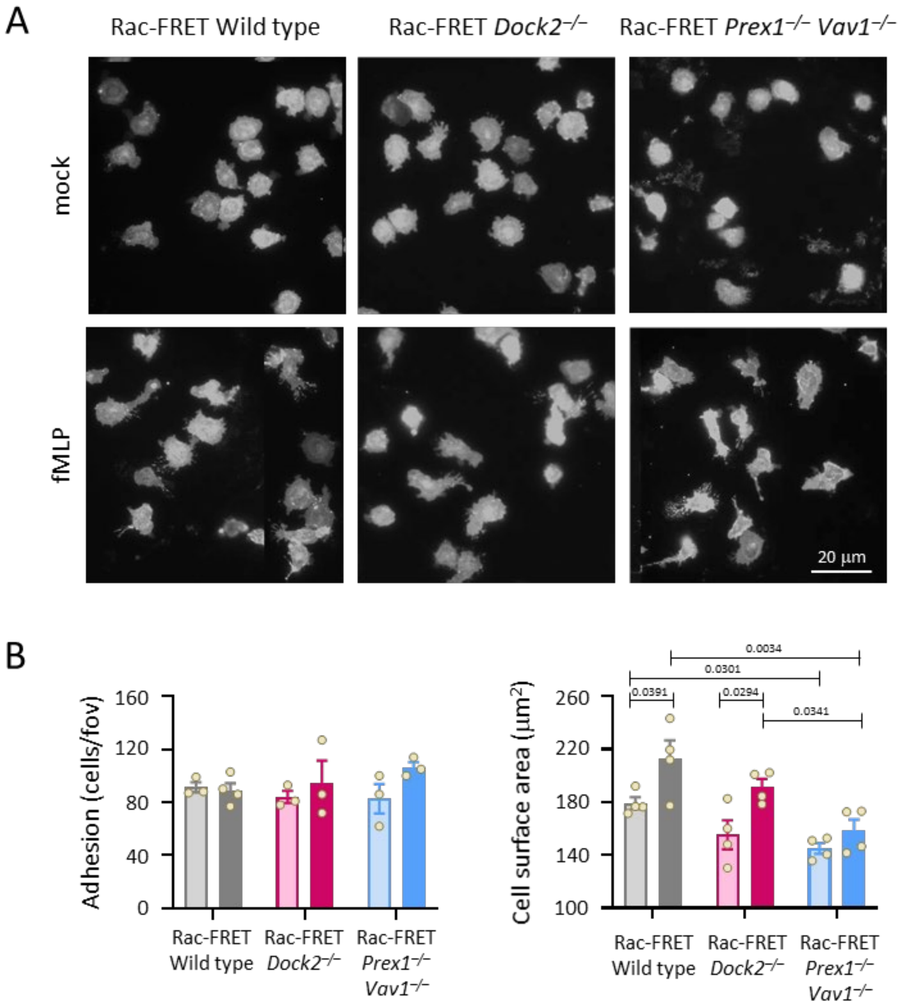

Supplementary Figure 1: Prex1/Vav1 but not Dock2 are required for neutrophil spreading on glass. Neutrophils from Rac activity FRET reporter mice (Rac-FRET wild type, grey bars), or from Rac-FRET mice deficient in the Rac-GEFs Dock2 (pink) or Prex1/Vav1 (blue) were primed with 50 ng/ml GM-CSF and 20 ng/ml TNFα before being plated onto glass coverslips and incubated for 10 or 25 min at 37°C in the presence (dark bars) or absence (light bars) of 0.75 µM fMLP. Adherent cells were fixed, stained with FTIC-Gr1 antibody, and imaged by wide-field fluorescence microscopy. (A) Representative images (FITC signal) from one experiment of cells stimulated with fMLP for 25 min, or mock stimulated, as indicated. (B) Images were analysed by Fiji for the number of adhering cells after 25 min incubation (left-hand panel) and for their surface area after 10 min (right-hand panel). Data are mean ± SEM of 4 independent experiments, with each dot representing the mean of one experiment, and with between 63 and 140 cells being analysed per genotype and condition in each experiment. Statistics are two-way ANOVA with Sidak's multiple comparison corrections.

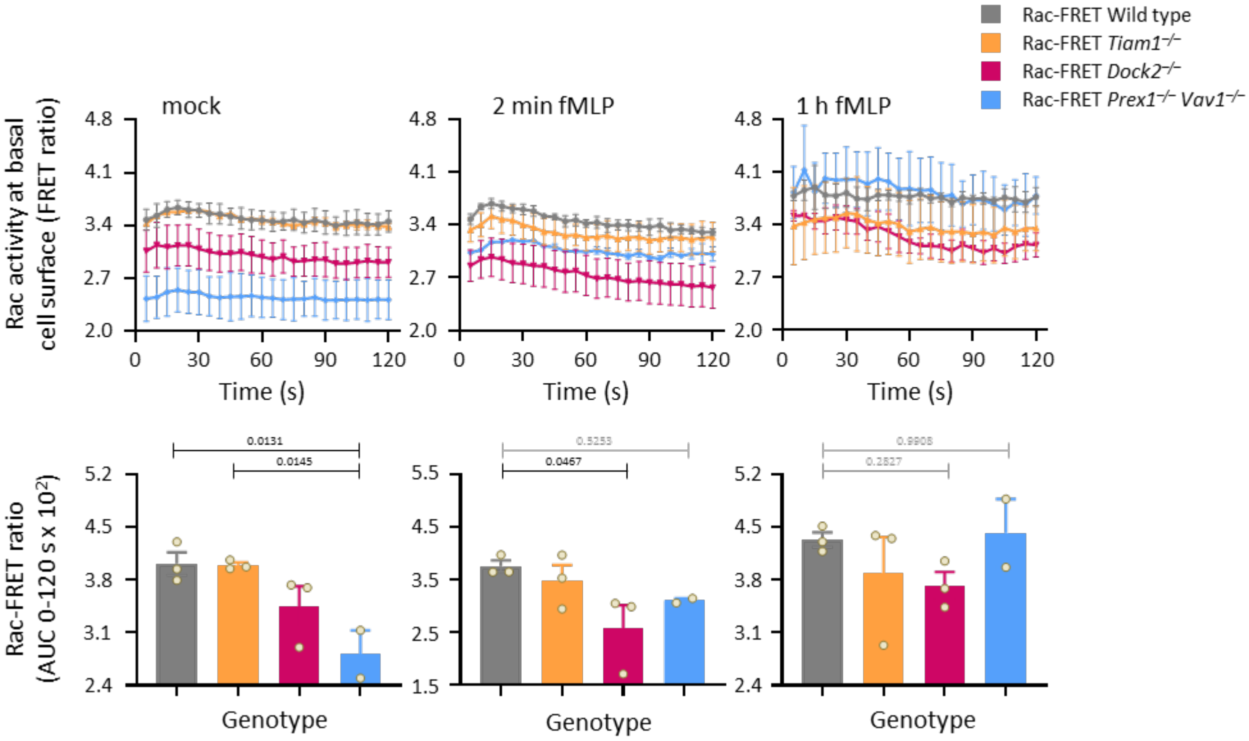

Supplementary Figure 2: fMLP stimulation reduces the involvement of Prex1/Vav1 and Dock2 in Rac activation during sustained neutrophil adhesion to ICAM1. Neutrophils from Rac-FRET wild type mice (grey symbols), or from Rac-FRET mice deficient in Tiam1 (yellow), Dock2 (pink) or Prex1/Vav1 (blue) were primed with TNFα and GM-CSF, plated onto ICAM1-coated ibidi slides, and allowed to adhere for 1 h at 37°C, and were then either mock-stimulated (left-hand panels) or stimulated with 0.75 µM fMLP (middle) for 2 min. During these 2 min, Rac activity was imaged by live-cell ratiometric TIRF-FRET microscopy at a frame interval of 5 s. Alternatively, cells were chronically stimulated with 0.75 µM fMLP for 1 h, in addition to the 2 min of imaging (right). Top panels: Rac activity (FRET ratio; mean ± SEM) on the basal surface of the cells, plotted over time. Bottom: Quantification of Rac activity as AUC integrated from the data shown above. Data are mean ± SEM of 3 independent experiments for Rac-FRET wild type, Tiam1$^{-/-}$ and Dock2$^{-/-}$ cells, and 2 experiments (mean ± range) for Prex1$^{-/-}$ Vav1$^{-/-}$ cells, with the different genotypes being compared directly within the experiments. Statistics are one-way ANOVA with Tukey's multiple comparison corrections.

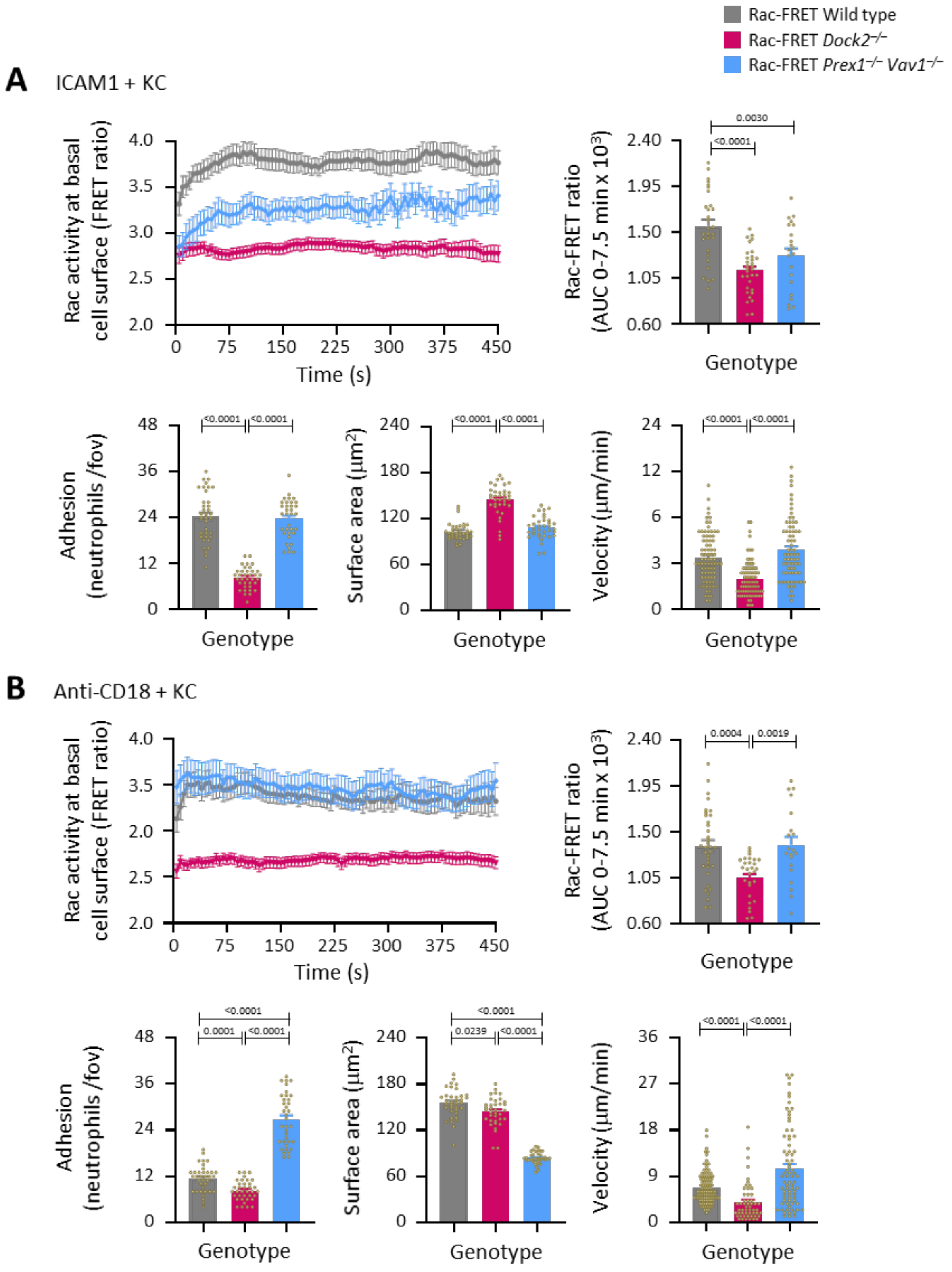
Rac-FRET Wild type
Rac-FRET Dock2−/−
Rac-FRET Prex1−/− Vav1−/−
A
ICAM1 + KC
Rac activity at basal cell surface (FRET ratio)
Time (s)
Rac-FRET ratio (AUC 0-7.5 min x 10³)
Genotype
0.0030
<0.0001
Adhesion (neutrophils /fov)
Surface area (µm²)
Velocity (µm/min)
B
Anti-CD18 + KC
0.0004
0.0019
0.0001
0.0239

Supplementary Figure 3: Dock2-mediated Rac activity correlates with neutrophil adhesion and chemokinesis on β2-integrin substrates in the presence of KC. Purified neutrophils from wild type Rac-FRET mice (grey symbols), or from Rac-FRET mice deficient in Dock2 (pink) or Prex1/Vav1 (blue) were plated onto (A) ICAM1-coated or (B) activating anti-CD18 antibody (M18/2) coated coverslips, and allowed to adhere at 37°C in the presence of 100 ng/ml KC. Rac activity (top panels) was imaged by live-cell ratiometric TIRF-FRET microscopy over 7.5 min, at a frame interval of 5 s. Cells that remained adherent for at least half the observation time were included in the analysis. Left: Rac activity (FRET ratio; mean ± SEM) at the cell basal surface over time. Right: Quantification of Rac activity as AUC of the data on the left. Data are mean ± SEM of cells pooled from 3 independent experiments for each surface, with direct comparison of all genotypes within experiments, except in (B) where Dock2$^{-/-}$ cells were from 2 experiments. The numbers of neutrophils tested were 29/35 wild type, 32/29 Dock2$^{-/-}$ and 21/20 Prex1$^{-/-}$ Vav1$^{-/-}$ cells in (A) and (B), respectively. Statistics are one-way ANOVA with Tukey's multiple comparison corrections. Adhesion and spreading (bottom panels) were measured in neutrophils fixed after 15 min. Images were analysed by Fiji for the number (left) and surface area (middle) of cells. Data are mean ± SEM of 36 fov/genotype; each dot represents one fov. Chemokinesis migration velocity (bottom right) was determined by widefield live-imaging of neutrophils from the moment they began to adhere. Data are mean ± SEM of 103 wild type, 53 Dock2$^{-/-}$, and 76 Prex1$^{-/-}$ Vav1$^{-/-}$ cells pooled from 3 independent experiments; each dot represents one cell. Statistics are one-way ANOVA on log-transformed data with Tukey's multiple comparison corrections.

Supplementary Movie Legends

Supplementary Movie 1. Rac activity during adhesion, spreading and polarisation of wild type Rac-FRET Wild type, Dock2$^{-/-}$and Prex1$^{-/-}$ Vav1$^{-/-}$ neutrophils. Rac-FRET Wild type (left), Dock2$^{-/-}$ (middle) and Prex1$^{-/-}$ Vav1$^{-/-}$ (right) neutrophils were plated onto glass coverslips and imaged by live-cell TIRF-FRET microscopy from the first point of contact with the slide for 10 min with a frame interval of 5 s to observe Rac activity during adhesion, spreading and polarisation. The movie shown is representative of 8 independent experiments for Wild type, 6 for Dock2$^{-/-}$, and 6 for Prex1$^{-/-}$ Vav1$^{-/-}$. The pseudo-colouring depicts high Rac activity (high FRET ratio) in white/red and low Rac activity in blue.

Supplementary Movie 2. Rac activity is highest at the leading edge of wild type Rac-FRET neutrophils migrating towards the chemoattractant in a micropipette chemotaxis assay. Neutrophils from wild type Rac-FRET mice were allowed to attach to glass coverslips and stimulated with a point source of 1 µM fMLP delivered from a microinjection needle placed ~20 µm 'west' of the cell, while being imaged by live-cell ratiometric TIRF-FRET imaging for 4 min, at a frame interval of 2 s. Half-way through the experiment, the needle was moved 90° 'south' to measure the ability of the cell to turn. The movie shows one neutrophil representative of 11 cells from one of 3 independent experiments. The pseudo-colouring depicts high Rac activity (high FRET ratio) in white/red and low Rac activity in blue.

Supplementary Movie 3. Rac activity is highest at the leading edge that makes contact with particles and at the phagosome of wild type Rac-FRET neutrophils. Wild type Rac-FRET neutrophils were primed with TNFα and GM-CSF, and were then incubated with IgG-opsonised 2 µm Fluoresbrite® yellow/green carboxylate microspheres, at a ratio of 10 particles/cell, while being live-imaged by ratiometric TIRF-FRET microscopy for 15 min, with frames taken every 5 s. Brightfield imaging was performed in parallel to visualise cells and particles at the same time. Left: Rac activity (FRET ratio). Right: brightfield imaging. The movie shows one neutrophil representative of 20 cells imaged from 3 independent experiments. The pseudo-colouring depicts high Rac activity (high FRET ratio) in white/red and low Rac activity in blue.
